# Food Safety Practices and Behavior Drivers in Traditional Food Markets in Ethiopia: Assessing the Potential for Consumer-Driven Interventions

**DOI:** 10.3390/ijerph22111645

**Published:** 2025-10-29

**Authors:** Ariel V. Garsow, Smret Hagos, Eric Djimeu, Carrel Fokou, Haley Swartz, Genet Gebremedhin, Bisaku Chacha, Elisabetta Lambertini

**Affiliations:** 1 Global Alliance for Improved Nutrition, Washington, DC 20005, USA; agarsow@gainhealth.org (A.V.G.); haleyswartz@gmail.com (H.S.); 2 Global Alliance for Improved Nutrition, Addis Ababa, Ethiopia; smret.hagos@sartconsult.com (S.H.); ggebremedhin@gainhealth.org (G.G.); 3 Results for Development, Washington, DC 20036, USA; edjimeu@r4d.org; 4 Department of Human Resource Economics, Faculty of Economics and Management, University of Maroua, Maroua P.O. Box 46, Cameroon; carrelfokou@gmail.com; 5 IPSOS, Dar es Salaam, Tanzania; bisaku.chacha@ipsos.co.tz

**Keywords:** food safety, food choice, behavior change, consumer demand, traditional markets, LMIC, Ethiopia, EatSafe, Feed the Future, foodborne disease (FBD)

## Abstract

Traditional food markets are a key node of resilient food systems worldwide. However, improper food handling and limitations in market structures may result in foodborne disease. This study assessed the decision-making of consumers and vendors in traditional markets to identify opportunities to reduce foodborne contamination and exposure. A cross-sectional survey of 150 consumers and 150 vendors was conducted in Sidama, Ethiopia, in July–August 2022 to investigate practices, behavior drivers, and enabling environment factors relevant to food safety. Descriptive statistics were used to summarize demographics and behavior variables, and the Poverty Probability Index for socioeconomic status. Women consumers were the primary deciders for what food to buy. Of those surveyed, 26% of vendors and 19% of consumers lived below $3.20 USD/day. Consumers choose to purchase food based on price, food quality, vendor personality, and food safety (assessed using visual and sensory cues). Vendors were unconcerned about foodborne disease (73%) and attributed business success to food quality, their personality, and offering discounts. Salience and demand for food quality, as well as trusted relationships, could be leveraged as business incentives for vendors to adopt food safety practices and to increase consumers’ preference for safer food and ability to identify it.

## 1. Introduction

Worldwide, foodborne illness is responsible for a considerable health burden. The Food Epidemiology Reference Group (FERG), a working group of the World Health Organization, estimated that 600 million people each year (approximately one in ten) become ill from food they eat [1,2]. Additionally, it is estimated that 420,000 people per year die of foodborne disease (FBD), 30% of whom are children below 5 years old. This burden predominantly affects low- and middle-income countries. Foodborne disease causes significant economic losses. In low- and middle-income countries alone, productivity losses due to foodborne diseases are estimated to amount to 95.2 billion USD, while 15 billion USD are expended for medical treatments [3].

FERG estimates also show that the group of African countries that includes Ethiopia (AFR E) has the second-highest per capita FBD burden in disability-adjusted life years, second only to the AFR D region, with most of the burden being due to diarrheal disease agents [1,2]. As Ethiopia transitions from a low-income country to a lower-middle-income country, it is experiencing rapid economic, demographic, and dietary changes. Despite these changes, the country continues to have a limited ability to control foodborne disease due to a lack of food safety legislation, enforcement capacity, and funding [3,4,5,6].

In Ethiopia, traditional markets (open-air markets where most people regularly buy and sell nutritious foods, including fresh vegetables and animal-source foods) often lack the infrastructure necessary for food safety and are largely unregulated by food safety authorities [7]. As in other African countries, market vendors in Ethiopia have been found to have gaps in safe food handling skills, while consumers have little representation in advocacy associations [4,8,9,10]. These factors heighten the risk of foodborne disease. As such, improving food safety in traditional markets represents an important opportunity to lower foodborne disease

Research tools on food choices related to food safety, both quantitative and qualitative, have been developed [11,12,13,14]. However, they primarily focus on practices and knowledge. These tools would benefit from the inclusion of behavior drivers such as motivations and incentives, self-efficacy, beliefs, and interpersonal dynamics that are crucial to sustained behavior change [15]. Improvements in tools to assess food safety choices are needed to adequately inform intervention design [16,17].

The US Agency for International Development (USAID) Feed the Future EatSafe (Evidence and Action Towards Safe, Nutritious Food) program tested interventions that seek to improve food safety by leveraging consumer demand for safe food, with a focus on nutritious foods sold in traditional markets. EatSafe in Ethiopia focused on kale, tomatoes, and lettuce, three fresh vegetables that are commonly sold and eaten in the community [18]. These vegetables, although a key part of a healthy diet, can be associated with foodborne disease [19,20]. EatSafe identified foodborne hazards, including *Salmonella* spp., in samples of fresh vegetables (tomatoes, lettuce, and kale) purchased from traditional markets in Ethiopia [21], highlighting the need for food safety improvements. Of the samples collected, 35% were found positive for generic *E. coli*, and 7% were positive for *Salmonella*. Levels of contamination varied by vegetable, with kale having the highest prevalence, followed by lettuce and tomatoes.

The objective of this study was to understand consumers’ motivations for food purchasing choices and vendors’ ability and motivation to carry out food safety practices, in order to inform the design of interventions to improve food safety in markets. Specific research questions included: (1) What are key food safety practices and their motivations, for market consumers and vendors? (2) Do consumers and vendors have a desire or motivation to buy and sell safe foods? (3) What drivers or obstacles should be considered when designing interventions? Results from this study may be used to inform the design of food safety interventions in traditional markets.

## 2. Materials and Methods

A cross-sectional survey was conducted in a mid-sized urban area in the Sidama region (South-western Ethiopia) from July–August 2022. The urban area was selected based on relevance to the Feed the Future program, the target foods of kale, lettuce, and tomato being widely consumed in the area, the city being large enough to have multiple markets, and the city having sufficient security to allow for the work to take place safely. Ethical approval for the study was received from the Sidama National Regional Health Bureau Public Health Institute Institutional Review Board (Number: DFI/3744/1).

**Figure 1:**
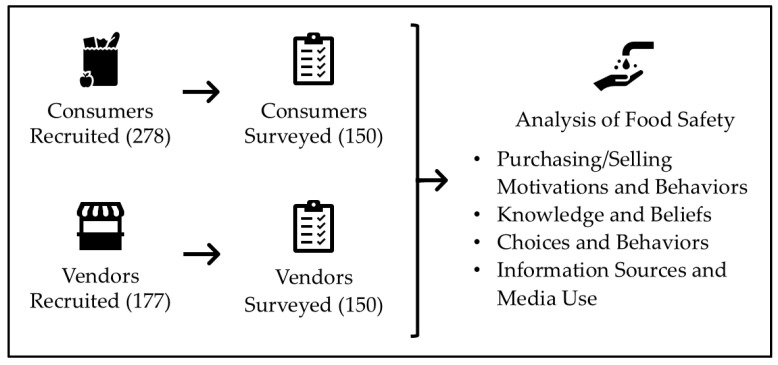
Study design.

Surveys were conducted with consumers and vendors from the study market (Figure 1). Respondent quotas (i.e., gender for consumers; gender and commodity sold for vendors) were determined using preliminary estimates of total market size. A total of 278 consumers were recruited and screened for eligibility at different locations in the market over multiple days and times of the day (including both main market days and less crowded days, and morning, midday, and afternoon times). Among eligible consumers, 150 were randomly selected following a gender-based stratification (65% women and 35% men, based on preliminary observations) to be surveyed. A total of 177 vendors were recruited and screened for eligibility on both main market days and less crowded days. Out of 177 vendors enrolled, 150 vendors were randomly selected within each commodity-gender stratum (observed vendor counts: 87% women and 13% men vendors; 15% kale, 76% tomato, 10% lettuce). Informed consent was obtained verbally upon enrollment and confirmed at the start of the questionnaire. Consumers and vendors could choose to be interviewed immediately or schedule the interview for a later day at a location of their choosing.

Consumers were eligible to participate in the study if they were over 18 years of age, had primary or shared responsibility for purchasing food for their household, and shopped for at least one key commodity in the target market at least once a month on average. Vendors were eligible to participate in the study if they were over 18 years of age, were a primary vendor in the shop, regularly performed key vending operations, and sold at least one of the key commodities (lettuce, tomatoes, or kale) at the target market at least one day per week for the past three months. One vendor per business was enrolled.

The consumer and vendor surveys included information on demographics; market behaviors; personal perceptions and motivations about food choices and food safety; and sources of food safety information (Figure 1; Supplementary Information S1 and S2). Surveys were administered in face-to-face interviews by trained enumerators in either Amharic, Sidama, Wolayita, or English. Data were collected and validated using SurveyCTO software (Dobility, Inc., https://www.surveycto.com; version 2.70.4), with the data entry interface programmed in Amharic and English.

The sample size for each of the two groups (150 vendors and consumers) was calculated to be able to estimate a prevalence of 0.5 (50%) for a belief or practice with a confidence level of 95% and a margin of error of 8% [22].

Statistical analyses were conducted using Stata release 17 [23]. Visualizations were produced using R version 4.2.0 for Windows [24]. Descriptive statistics were used to describe participatant demographics; market behaviors; perceptions and motivations; and sources of food safety information. The mean and standard deviation (SD) of numeric variables were calculated. The proportion for categorical variables was calculated. T-tests were used to compare means between genders (men and women) for numeric data. Fisher’s exact tests were used to determine significant differences in proportions by gender for all categorical variables with binary answer options. Chi-square tests were used to evaluate the gender-based differences for categorical variables with more than two answer options. Statistical differences between men and women are stated (*p* ≤ 0.05). Results are summarized across all vendors (N = 150) or consumers (N = 150) unless otherwise stated.

An Ethiopia-specific Poverty Probability Index (PPI) was used as an indicator of socio-economic status (SES; Supplementary Information S3) [25]. The PPI is based on ten indicators of wealth/poverty. In the original version of the Ethiopia PPI Scorecard, the final question was about the number of machetes (gejera) owned. As this question was identified as potentially sensitive by GAIN staff and implementation partners, a new Scorecard was developed and calibrated in coordination with the developers of the PPI (Innovations for Poverty Action, IPA). For each respondent, the PPI is scored on a 100-point scale, where higher values indicate higher SES (Supplementary Information S3). PPI values were converted to probabilities of poverty using tables provided by the PPI developers. Two poverty lines were used: the Ethiopian National Poverty Line (NPL) of 7184 ETB/year (0.38 USD/day) and the international poverty line of 3.20 USD /day developed by the World Bank.

Potential outliers for each continuous variable in the dataset were identified using the z-score. Observations with an absolute value of the z-score greater than 3 were considered potential outliers and assessed considering the study context [26]. For example, answers of “100” for the number of suppliers a vendor has were omitted from the analysis for this variable.

## 3. Results

### 3.1. Respondent Demographics

A total of 300 respondents, 150 consumers and 150 vendors, were interviewed (Table 1).

**Figure 2:**
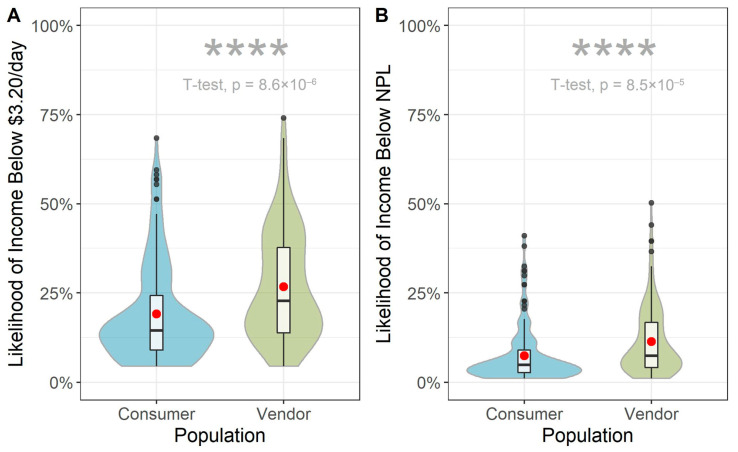
Probability of poverty according to (**A**) international 3.20 USD /day poverty line; (**B**) Ethiopia-specific NPL of 7184 ETB/year (0.38 USD/day). Red points correspond to the group mean probability of poverty. Asterisks signify statistical significance with *p* ≤ 0.0001.

Among vendors, most respondents were the owners of the shop (93%). Over half of the interviewed consumers and vendors (67% and 58%, respectively), were the head of their household. Nearly all surveyed individuals had access to electricity (98% vendors and 99% consumers, respectively). To characterize the socioeconomic status of respondents, variables related to household assets were used to compute the Poverty Probability Index (PPI) with the international poverty line of $3.20/day and a specific Ethiopian national poverty line (Ethiopia NPL) of 7184 ETB/day. At the $3.20/day poverty line (Figure 2A), the mean probability of poverty was 28% for consumers and 34% for vendors (±14% SD; median 25%, IQR: 16–35%; Figure 2A). Using the Ethiopian NPL, estimates were lower: 11% and 16% for consumers and vendors, respectively (±10% SD; median 13%, IQR: 8–20%; Figure 2B). These percentages correspond to the proportion of the population estimated to be living in poverty. While a substantial proportion of the population would be considered impoverished according to the international USD 3.20/day poverty line, the proportion of the population considered impoverished by national standards is much lower. There was no difference observed across genders, but the probability of poverty was significantly higher for vendors than consumers across both poverty lines (*p* < 0.0001).

### 3.2. Consumer Survey

This section reviews consumer behaviors and motivations that can inform the design of food safety and nutrition interventions. These include food-related gender roles in the household; food shopping patterns at the market; key characteristics that consumers seek when deciding which food to buy and which vendors to buy from; signals/cues used to identify unsafe food; interactions and communications with vendors; and beliefs or perceptions related to food safety in the market.

#### 3.2.1. Consumer Food Purchasing Motivations and Behaviors

The majority of respondents had primary responsibility for going to buy food (73%; 44% women and 29% men). This was expected since respondents were recruited at the market. Women were the primary decision maker for what food is purchased (57%). Shoppers primarily purchased plant-source food at this market (Table 2).

Most consumers purchase food from a local traditional market (89%), compared to other locations like a supermarket (4%). The main reason that respondents visit the study market is its convenient location (67%). Study respondents were satisfied with the market overall (85%) as well as with the options of vendors (84%). Over half (57%) of respondents felt secure at the market (physical or personal safety, “not being worried about harassment, theft, or assault”), and 18% felt somewhat secure.

Most respondents had been shopping at the study market for more than three years (91%). Of consumers, 74% visited once a week, and 21% visited more than once a week. The times that study respondents most frequently visit the market are in the morning between 8 AM and 12 PM (36%), or in the early afternoon between 2 PM and 5 PM (29%). During a typical shopping trip, respondents report having plenty of time (81%) to shop and visit around 4 vendors (SD ± 2) per visit. Consumers generally know what they need (80%), and most do not have a written shopping list (only 32% do have a list). The main purpose for consumers to visit the market is to buy food (93%) instead of other activities such as talking to friends (6%). Additionally, most consumers reported never talking to other customers about food purchasing decisions (37%). Bartering with vendors at the market was not common (25%).

**Figure 3:**
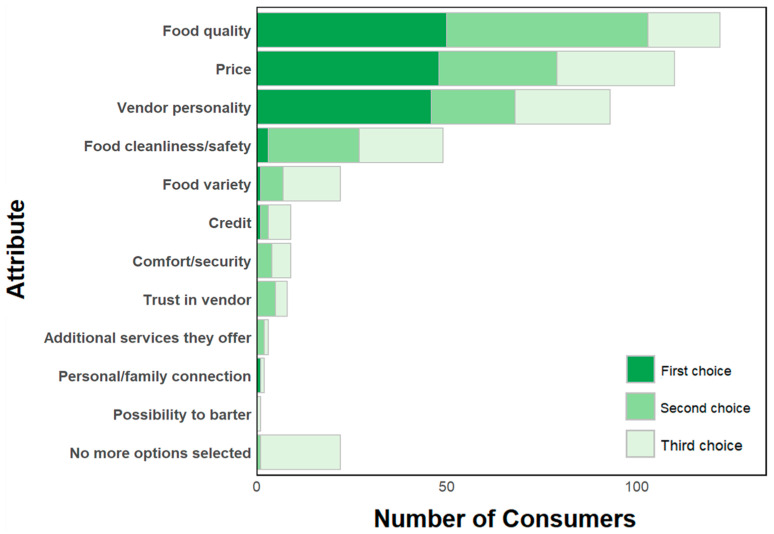
Reasons consumers choose to purchase food from a particular vendor. (This question gave the option for respondents to provide three answers. Answers were recorded in the order of being mentioned. “First choice” means that this attribute was mentioned first.

Consumers choose a vendor from whom to buy based on food quality, price, vendor personality, and perceived safety of the food being sold (Figure 3). Regularly buying food from the same vendor is common. Consumers tend to visit the same shops for vegetable purchasing (52%). Of the consumers that compare vendors (77%, N = 116), when asked how often they compare different commodities, they reported to compare vendors of leafy greens and tomatoes the most frequently (at least sometimes; 65% and 77%, respectively), but much less frequently for other commodities (35% for roots/tubers, 29% for legumes, and 4–9% for grains, poultry, eggs, or milk/dairy products). Consumers compare about 3 vendors before deciding to purchase.

Half of the interviewed consumers were satisfied with the food they bought at the market, while about one-third were very satisfied. The most important attributes used to select food items were freshness (74%), safety (10%), nutritional content/healthiness (6%), and price (5%). Answers to the most important attributes for food differed when asked about the most important attributes of food for their small children (less than five years old). In this case, the most important attributes were freshness (52%), safety (15%), healthiness/nutritional content (13%), and a balanced or varied diet (7%). For the majority of households, children under five years old never or only occasionally consume the same food as the rest of the household (56%, N = 62). Of the consumers interviewed, half of them were completely satisfied with the healthiness of their household food.

#### 3.2.2. Consumer Food Safety Knowledge and Beliefs

Respondents defined “food safety” in a variety of ways. Overall, the top five definitions of food safety that consumers gave included maintaining the cleanliness of the food; healthfulness/nutritional value of food; maintaining the quality of the food; food that has not spoiled; and food that is free of germs and bacteria. Individuals stated that signs of unsafe food include spoilage or rotting (31%), changes in flavor (12%), changes in texture (11%), or infestation by pests or insects (8%).

About half of consumers did not believe that individuals could get sick from eating kale, lettuce, and/or tomatoes (Table 3). Overall, consumers believe that vendors sell safe food, but they acknowledged that there were differences in food safety between vendors.

Only 9% (N = 14) of consumers reported having had a foodborne illness in the year prior to the interview. The foodborne diseases (FBDs) that individuals surveyed were most concerned about included diseases caused by microbial pathogens, including typhoid fever or amoebas.

#### 3.2.3. Consumer Food Safety Choices and Behaviors

Overall, food quality and price were the most important attributes that consumers look for when deciding which vendor to buy from. Food safety was reported less often (Figure 3). In addition, for most consumers, market cleanliness was not a reason for choosing which market to buy from (76%). Over half (58%) of respondents had neutral opinions about market cleanliness. These consumers perceived the market as “not too clean but not too dirty.” The reasons the remaining respondents gave for the market being very dirty or dirty (24%; N = 123) included waste, improper disposal of leftovers, mud, and vendors not cleaning their area after selling products.

**Figure 4:**
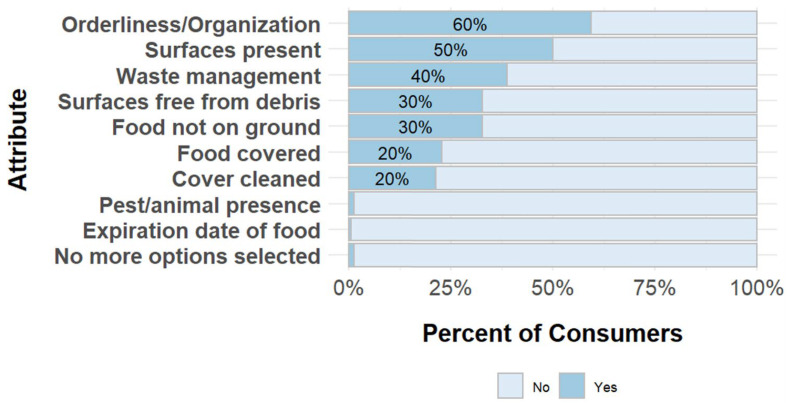
Factors consumers consider when evaluating the cleanliness of vendors.

However, consumers did feel that it was important that vendors had good personal hygiene (97% stated it was highly or very highly important). Consumers evaluated cleanliness or hygiene of a vendor by their orderliness or organization, open surfaces on a vendors’ counter, and if these surfaces were clean (Figure 4). Actions that consumers stated that vendors can take to improve food safety included procuring good quality product/maintaining product quality (37%), and cleaning the area in and around their shop (28%). Attributes that consumers mentioned to assess the hygiene behaviors of vendors included that vendors take care of their shop (shop cleanliness), knowing where the produce came from, and knowing how the produce was handled.

A majority of respondents stated that they would not purchase a food item if they were unsure about its safety (76%). Consumers would prefer to buy from vendors with a food safety certificate or license (Table 3).

Consumers mainly express the demand for specific food products or attributes through purchase choices, and sometimes through verbal communication. For example, while most consumers reported they have never stopped buying from a particular vendor (53%), 39% said they occasionally or sometimes do stop. The most common reasons for stopping to purchase from a vendor included food quality, price, and the vendor-customer relationship. Most consumers (76%) reported never talking to a vendor about food quality. Of the remaining 24% (N = 36) who discussed food quality with vendors, 64% were likely to initiate conversations about food safety. When discussing particular food items with vendors, tomatoes and leafy greens were the most often discussed (86% and 67%, respectively; N = 36). Most consumers (71%) never complained to vegetable vendors about their food purchases. Of the remaining 29% (N = 44) that did complain about food purchases, characteristics discussed during a complaint included options for different varieties of products, blemishes, and sizes and shapes of vegetables (mentioned by 32–41% of this subgroup). These findings suggest a minority of consumers are comfortable voicing their concerns to vendors.

Consumers noted that they adopt some risk reduction measures at home during food preparation. For instance, nearly all respondents (99%) wash kale. Additionally, kale is rarely eaten raw (63% cook or boil it before eating).

#### 3.2.4. Consumer Food Information Sources and Media Use

Most consumers reported owning a cell phone (95%). Among those who own a cell phone, 57% have a basic or feature phone, and 61% own a smartphone. All men surveyed personally owned a mobile phone, while 8% of the women did not (*p*-value = 0.05). The mean number of cellphones in the household was 2.6 (SD ± 1.6). Most individuals owned a television (79%), while slightly fewer owned a radio (61%).

About half of the surveyed consumers had access to the internet, primarily accessed via smartphones (96%, N = 63 of 66). The internet was accessed primarily at home (82%, N = 54 of 66). Facebook (88%), Telegram (68%), and YouTube (53%) were the most regularly used social media platforms by those who had internet access (N = 66).

Consumers trusted medical professionals (92%) to provide reliable information on health issues. If they wanted to obtain information on food safety, consumers would consult medical professionals (67%), friends or family (63%), food packaging or labels (47%), experts in the media (33%), and internet/social media (29%; Table 4).

A majority of consumers (90%) reported that they had no specific issues that they would like to know more about regarding food in the last year. Questions that the remaining respondents had about whether a certain food was safe or unsafe to eat included: “How do worms form in the stomach?”, “What causes food to spoil or become contaminated?”, “What are the agents that contaminate foods?”, “What causes typhoid?”, “How do you achieve a balanced diet?”, “How safe are packaged foods?”. Of those who sought out information to answer these questions (N = 6), all of them spoke to medical professionals; two of the six additionally reviewed newspapers, television, or radio; and one additionally searched on the internet.

**Figure 5:**
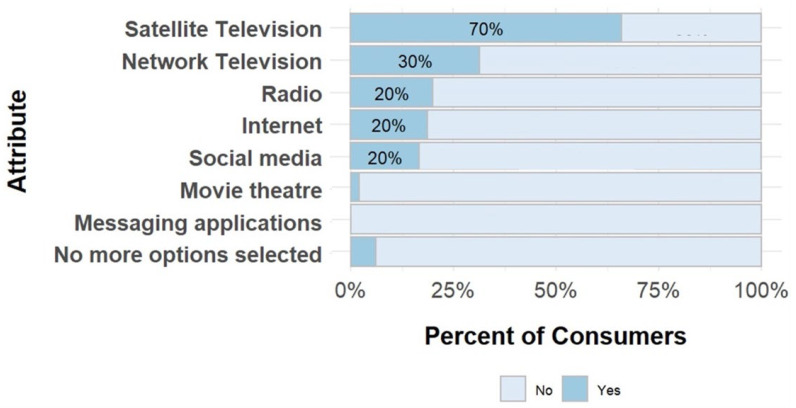
Source of media used for entertainment for consumers.

Consumers use different media channels for entertainment purposes (Figure 5). Satellite TV was the most frequently mentioned media type (70%), followed by network TV (30%), and radio (20%), all of which were accessed daily.

### 3.3. Vendor Survey

This section reviews vendor behaviors and motivations that can inform the design of food safety interventions. Topics in the survey included what products vendors sell; how vendors choose suppliers; how often vendors purchase new batches of food; actions that vendors take to promote purchasing of their products; communication among vendors and consumers; reasons vendors think customers complain; cleaning practices of vendors; where unsold food is kept; what sources of information and media vendors use.

#### 3.3.1. General Food Vending Practices

Most of the vendors surveyed did not own land (93%), cultivate any food crops (94%) or own livestock (84%). The majority (95%) did not produce the commodities they sold in the market. Three quarters of vendors sold tomatoes, while 18% sold kale and 10% sold lettuce. Half of vendors (58%) changed the commodities they sold by season. The primary reason for this change in types of food items sold was due to the quality of the food varying during rainy (June, July, and August) and dry seasons (December, January, and February; reported by 35% of vendors).

**Figure 6:**
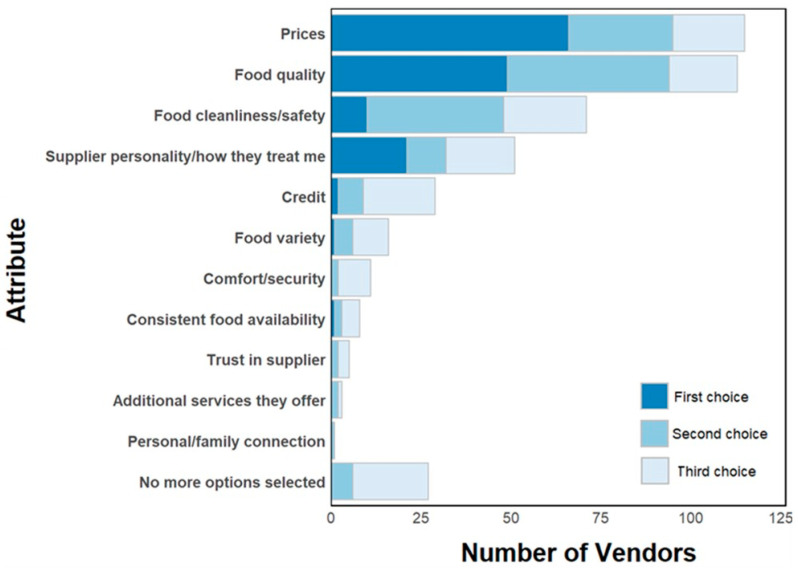
Reasons that vendors purchase food from a particular supplier.

On average, vendors source food from nine suppliers, although this number varied (SD ± 23). Vendors predominantly purchase food from wholesalers (89%), and these vendors often (78% of the time; SD ± 28) repeatedly purchase from the same wholesaler(s). Vendors chose suppliers by their price, food quality, food cleanliness/safety, and how suppliers treat them (Figure 6). If they wanted to change suppliers, most respondents (81%) indicated that they could do so.

Vendors reported receiving new batches of kale daily (26%, N = 7 of 27), new batches of lettuce two days per week (47%, N = 7 of 15), and new batches of tomatoes three times per week (33%, N = 37 of 114). Vendors generally brought food to the market themselves (75%). Women had suppliers bring food to their shop more often than men (27%, N = 34 of 128 and 9%, N = 2 of 22, respectively), which potentially reflects differences in access to equipment or a vehicle to transport products. There was a significant difference between how women and men transport food to the market (*p*-value = 0.01).

**Figure 7:**
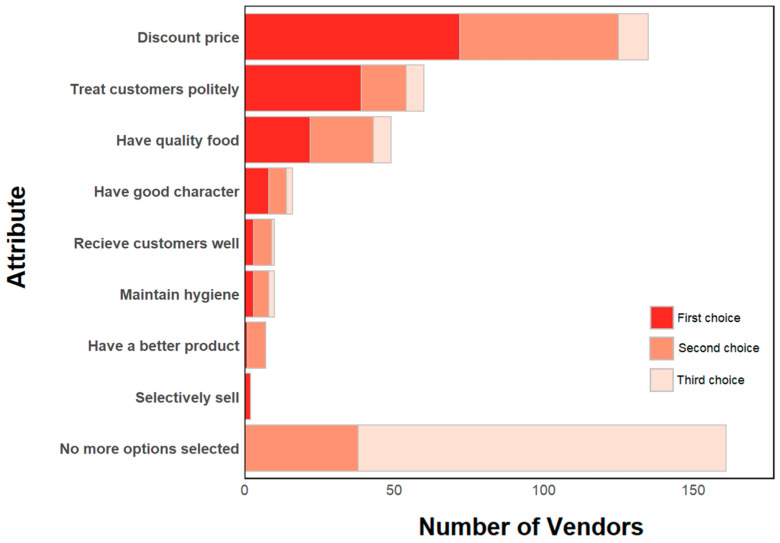
Actions that vendors take to promote purchasing.

Most vendors are satisfied with their experience selling at the market (79%) and feel that they are physically safe when selling food at the market (81%). Most shops consisted of a tarp on the floor (40%) or a wood structure (25%). On average, vendors have been selling at this market for 8.1 years (SD ± 7.0), with women (Mean = 8.5, SD ± 7.0) having been vendors at this market for more time on average than men (Mean = 6.0, SD ± 6.7). This difference is significant (*p*-value < 0.05). All vendors surveyed only sell produce at the target market. Additionally, working at the shop is the primary income-generating activity for most vendors (97%). There generally are no additional staff working at the shop (76%). Most shops are open year-round (98%). Per day, vendors on average sell to 9 customers (SD ± 10), with an average of 4 customers being regular customers (SD ± 3). Men were found to sell to more customers (mean = 14) on average than women (mean = 9, *p*-value = 0.01). Vendors perceive that the reasons that customers choose to purchase food from their shop include the quality of the food, their personality, and giving discounts on their products. Many vendors report using only one or two actions to promote consumer purchasing of their products, including discounting the prices of their products, treating customers politely, and having quality food (Figure 7).

Overall, vendors felt supported by other vendors. If vendors need help doing something in their shop, they trust that other vendors will help them (58%). Reasons that a vendor did not trust that other vendors would help them included vendors acting independently and the negative perception of asking for help.

#### 3.3.2. Vendor Food Safety Knowledge and Beliefs

Overall, vendors thought that the market was moderately clean (Mean = 3.3 on a 5-point scale, SD ± 0.9). Of the 21 vendors that rated the market as dirty or very dirty, improper waste disposal and rain were most frequently mentioned as primary reasons.

Vendors are generally not worried about foodborne disease (73%). Only 7% indicated they were worried about bacteria. While vendors generally do not perceive food as risky, they desire information on the topic. Several vendors expressed interest in learning more about bacteria and how negative health effects, such as diarrhea, can be related to contaminated food. Only 3% of vendors reported that they or someone in their household experienced a foodborne illness in the last year, compared to 9% of consumers.

#### 3.3.3. Vendor Food Safety Choices and Behaviors

Vendors felt confident in their ability to find suppliers that sell high-quality food and choose safe foods from these suppliers (“agree” and “strongly agree” comprised >90% for all responses). Most vendors (62%) also said that they would spend more time and money selecting safer foods. The variation in answers was slightly greater amongst women than men, with women having a wider range of answers (Table 5).

Vendors are satisfied with their current suppliers (Median: 5.0, SD ± 0.8) due to the quality, price, and cleanliness of the food that they sell. Suppliers and vendors care about the quality, price, and safety of food. Vendors noted several visual cues to identify signs that a batch of food may be unsafe, including signs of rotting or spoiling, or vendors’ unhygienic practices.

Vendors agree that customers tell them when they are satisfied with the food they provide (Median 4.0, SD ± 0.6). Men reported that customers tell them they are satisfied with the food they provide more often than women (Median for men: 4.5, SD ± 0.5; Median for women: 4.0, SD ± 0.6).

**Figure 8:**
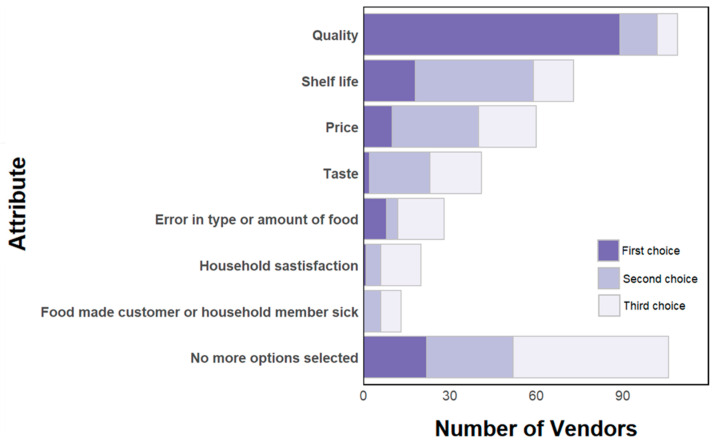
Vendors perception of customer complaints.

Verbal communication on food attributes is uncommon. Vendors reported that customers infrequently or never ask where their food comes from (92%). Vendors generally do not have conversations about the safety of the food that they sell with consumers (61%). The quality or variety of tomatoes is most commonly discussed (45%). Out of the vendors that had customers complain, the most common complaints were about the quality, shelf life, price, and taste of their products. A small number of vendors stated that they have heard customers complain about their food making a customer or household member sick (Figure 8). Similarly, vendors reported that they do not often or never have conversations with suppliers about food safety and quality (82%).

Nearly all (97%) of vendors agreed or strongly agreed that they were proud of the quality of the food they sold. Most vendors were satisfied with overall shop operations (79%). While over half of those surveyed agreed that there were specific rules for preserving food safety and shop cleanliness, about one-third disagreed (Table 6).

Nearly all vendors (97%) reported that they have not made changes to the structure of their shop or the way they sell food in the past year, but they have taken actions to keep food safe. In the past year, vendors acquired towels, knives, umbrellas, and fly whisks to keep food safe. Vendors reported sweeping/cleaning their shop daily (58%) as well as washing the food that they sell daily (19%). On average, vendors said that they wash their hands 3.3 times per day (SD ± 1.9). Additional actions vendors performed to keep food safe included using a fly whisk to get flies off of food (15%), cleaning food with a cloth (12%), and covering food from the sun with a shade or a polypropylene fabric (12%).

**Figure 9:**
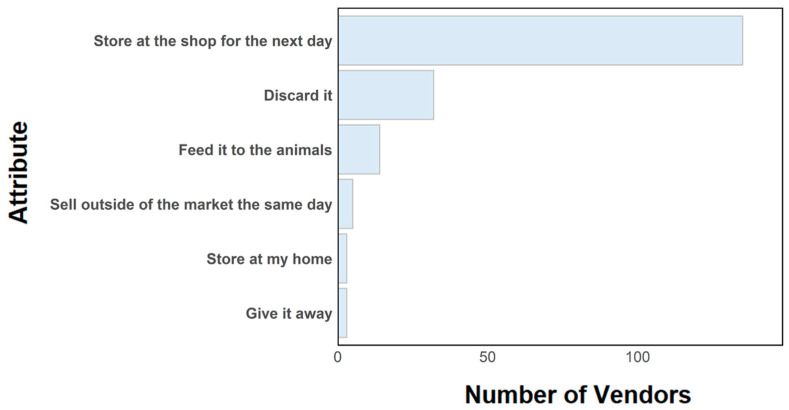
Actions vegetable vendors take with unsold food.

Unsold food was generally kept at the shop for the next day (90%; Figure 9). The majority of the vendors surveyed did not have a refrigerator in their household (83%, N = 125 of 150). Alternative ways vendors store food include covering it or putting it in a crate.

#### 3.3.4. Vendor Information Sources and Media Use

The households of most vendors own at least one cell phone (97%), a television (70%), a satellite dish (69%), and a radio (58%). Of the vendors surveyed, 73% of vendors owned at least one mobile (non-smart) phone and 18% owned a smartphone.

The majority (91%) of the vendors surveyed do not have access to the internet. Of those that do, a smartphone was the most frequently mentioned device respondents utilized to access the internet (86%, N = 12 of 14 who have access to the internet) followed by a mobile tablet and a desktop computer (7%, N = 1 of 14 each). Among the social media platforms, most of the vendors regularly used Facebook and Telegram (64%, N = 9 of 14 and 64%, N = 9 of 14, respectively). The most common media channel vendors used for entertainment included network and satellite television (74% and 33%, respectively).

Of the vendors who watched network or normal television, most vendors watched it daily (87%, N = 97 of 111). In contrast, of the vendors who watched satellite television, some watched it daily (41%), but a larger percentage watched it two or three times a week (47%). When asked about the types of entertainment content viewed, vendors most frequently mentioned TV series or soap operas (76%). Women (63%) sought out this content more than men (13%).

Vendors trust medical professionals to provide reliable information about health issues (95%). To determine the safety of food, vendors would predominantly consult friends or family (70%) and medical professionals (71%).

## 4. Discussion

This study revealed important elements to consider when designing food safety interventions involving consumers and vendors in traditional markets, and possibly in other food environments in Ethiopia. Several factors may provide incentives or constraints for adopting purchasing and handling behaviors that promote food safety, including the following: consumer shopping practices and loyalty; consumer food choice drivers; perceptions and cues of safe/unsafe food; costs and prices; information gaps and demand; vendor reputation; logistical challenges; and gender roles.

### 4.1. Food Purchasing Motivations and Behaviors

Limited food safety intervention studies have considered the relational dynamics between consumers and vendors [15]. In this study, it was found that consumers are loyal customers, as many of them have been shopping at the market for more than three years. Consumers’ choice of which vendor to purchase from within the market is influenced by food quality, safety, price, and the personality of the vendor. Because consumers commonly buy food from a particular preferred vendor, and sometimes compare vendors, interventions could leverage these relationships. Vendors could see an incentive to improve their food safety practices, either by being favored by consumers who compare shops and/or by enhancing trusted relationships with regular customers. For this incentive to drive behavior change in vendors, consumers need to perceive, value, and reward food safety improvements through patronage or by increasing the vendor’s reputation in the community.

### 4.2. Food Safety Knowledge and Beliefs

Findings from this study demonstrate a gap in risk awareness as well as a demand for information related to food safety. Consumers generally did not believe that individuals can become sick from consuming kale, lettuce, or tomatoes. Similarly, vendors were not generally concerned about foodborne diseases. In practice, proper sourcing and handling of fresh vegetables are crucial to prevent foodborne disease [20,27]. However, both vendors and consumers expressed an interest in learning more about how negative health effects such as diarrhea can be related to contaminated food. Increasing the salience of both the positive impacts of food safety and the negative impacts of FBD may be important when trying to motivate consumers to explicitly pay attention to food safety cues while shopping. However, awareness or information are generally not sufficient to lead to action without either an emotional reward and/or a rational incentive. It has been found that a broad range of behavior drivers needs to be accounted for in interventions [11,18]. Additional opportunities, in addition or in alternative to direct messaging on food safety, include embedding food safety cues and objectives within broader characteristics that are already salient and important to consumers, such as food quality (e.g., high quality food should also be clean, fresh, free of flies, with an unbroken surface) or the trustworthiness of a vendor (e.g., a trustworthy vendor keeps their shop clean, tidy, and without pests).

### 4.3. Consumer Food Safety Choices and Behaviors

Food safety is usually not a factor of primary salience for consumers when selecting a vendor and buying food. In this study, it was found that price and perceived quality (which may indirectly include safety attributes) are primary purchase factors that both consumers and vendors agree upon. Other studies in markets and with street food vendors across multiple geographies have also shown the dominance of these factors [11,28,29]. Although perceived food safety is not commonly reported as an explicit purchase factor, other characteristics such as shop neatness and vendor trustworthiness may implicitly be associated with food safety practices and could be leveraged in tandem with more explicit food safety cues to inform consumer preferences [12,29,30,31].

Consumers and vendors have different opinions on the safety of food sold at the market. Vendors felt confident in their ability to find suppliers that sell high-quality food and to choose safe food from suppliers, while consumers stated that differences exist in the safety of food sold by different vendors. When specifically prompted about assessing food safety and shop hygiene, consumers primarily relied on visual cues. An opportunity exists to enhance the visibility of these cues (or introduce new ones) and increase the perceived association between cues and benefits for the consumer. For instance, food safety practices could be made visible or highlighted to catch the attention of consumers via colorful tools (e.g., cleaning rags, waste bins, washable surfaces, or aprons), shop or food brands, or food labels. This strategy has been tested by EatSafe and other programs [32,33,34].

Price is a key factor that influences the food purchasing decisions of consumers when selecting a market, a vendor, and food. Interventions to increase food safety need to be sensitive to price thresholds and consumers’ willingness to pay for food of different quality. This is particularly important for low-SES households. Since most low-income consumers are generally not willing or able to pay a premium for safer food [35,36]. Cost and price increases should be minimized, or possibly managed for a combination of attributes that consumers value (e.g., food quality, convenience, and bulk discounts). The literature on willingness-to-pay for safer food is rapidly evolving and includes circumstances where it might be leveraged as an incentive [37,38,39,40]. Education programs might increase awareness of the cost of foodborne illness (e.g., loss of income or cost of medical interventions), as a way to increase consumers’ willingness to pay for safer food. However, price is likely to remain a top purchase choice factor. Additionally, financial considerations for different consumer segments need to be considered. In this study, a sizable percentage of respondents lived below the International Poverty Line of $3.20 USD/day (26% of vendors and 19% of consumers) Even in cases where a small food price increase to cover the costs of supplies for a new food safety practice may be within consumer willingness to pay thresholds, safeguards to mitigate the unintended consequence of such increases should be included in program design.

The cost of interventions for vendors also needs to be carefully considered. For many vendors, selling food at the market is their main livelihood. Ideally, interventions would result in minimal operating cost increases for the vendor while attracting more customers, thus increasing sales. Minimal cost increases associated with food safety interventions would incentivize individual vendors to continue implementing safer food practices and potentially motivate other vendors to adopt them. Competition among vendors seems to be accepted, suggesting there could be a role for interventions focusing on individual stalls/vendors. However, market-based programs involving many or all vendors could also leverage the values of collaboration and equity. For example, structural improvements or services such as the provision of potable water, waste management, or raised stalls could be more efficiently secured, and potentially more easily accepted, via collective investments. Vendor demand for and willingness to invest in such market-wide improvements, while unlikely to be motivated by food safety alone, could be driven by other business incentives (e.g., better consumer access to their stall, better working conditions, or less food spoilage and waste). Food safety design could then be embedded in these upgrades. However, investing in improvements that benefit all vendors might reduce incentives for individual vendors to “stand out” from the competitors by investing in their stalls. The balance between competitive and collaborative dynamics among vendors has been observed in other contexts and needs further research [30,41,42]. Additionally, interventions should be aware of whether vendors are licensed to sell products in the market, as their official vs. unofficial status could influence the vendors’ willingness to participate in activities, costs incurred, and dynamics among vendors.

Overall, evidence is lacking on the perceived connection between food safety practices, reputation, and business success for vendors in LMICs [43]. The reasons why vendors may decide to practice behaviors that promote food safety are often a combination of multiple factors including what customers perceive as desirable [44]. For vendors, awareness of potential reputation loss due to customer reactions to becoming sick from the food they sell may provide an incentive to adopt improved practices. Situations where vendors may be more incentivized to adopt food safety practices include if the illness can potentially be traced back to their shop, if the cause of the illness is considered to be under the control of the vendor (if the vendor can take action to prevent contamination), and if the likelihood of occurrence is high (not just a low-likelihood hypothetical). The risk of reputation damage was also found to be an incentive towards food safety-promoting behaviors for vendors in northwest Nigeria [44]. Conversely, a positive reputation for clean and safe food may be perceived as attracting and retaining customers, as found in a study in Vietnam [45].

Additionally, logistical challenges affecting vendors could be leveraged to increase their motivation and self-efficacy for safer food handling. In this study, it was found that the majority of surveyed vendors store excess food at their shops overnight in order to try to sell it the next day. Concerns regarding loss of income due to product losses are common and have been observed for vendors in other countries, including in northern Nigeria [46]. Unsold food could be a potential food safety concern, depending on how the food is stored. Other practical challenges identified in studies from a range of countries include unclear rules and regulations, lack of tools or infrastructure (e.g., water) necessary for safety practices, lack of a market’s master plan (e.g., to separate commodities), and lack of training [43]. Supporting vendors in solving these challenges would likely result in food safety benefits.

Gender considerations should be accounted for in interventions. For both consumers and vendors, important differences in roles and social norms were demonstrated between men and women. Most fresh vegetable vendors at the market were women. Women vendors, on average, had been vending at the market for a longer time than men. Women and men were found to have different ways of transporting food to the market, with women having suppliers bring food to their shop more commonly than men. Women were also found to be primarily responsible for food preparation in the household, which could mean increased familiarity with the nuances of food quality and food handling practices. As a consequence, behavior change targets, motivators, and channels for food safety interventions may need to be different between women and men.

### 4.4. Food Information Sources and Media Use

It is important to understand how information is shared and received. Both consumers and vendors in this study rarely, if ever, talked about food quality and safety during market transactions, suggesting that verbal communication was not a common way to express demand. Vendors also did not commonly discuss food quality or safety with their suppliers. At the same time, vendors perceived their fellow vendors as collaborative and available to help each other if there was a need, suggesting openness to interaction. When asked where they obtain information about food safety, both groups stated they would consult family, friends, and medical professionals. The important role of community and family members in sharing food safety information was also noted in other contexts [12]. Interventions could leverage these communication channels to increase the salience of and demand for improved food safety practices.

### 4.5. Study Limitations

This study has limitations that should be considered when interpreting the results. First, it is a cross-sectional survey conducted in one market in a mid-size city in the Sidama region in Ethiopia, which may limit the generalizability of findings. A comparison across countries was not in scope for this study. However, similar results were found in a study conducted by the research team in Nigeria [47]. Second, a convenience non-random sample may introduce biases by selecting certain respondents and not others, e.g., those with less time constraints or those who are comfortable providing their personal information (e.g., registered vs. unofficial vendors). In addition, structured surveys have limits in the range and nuance of questions that can be effectively answered in a short time. EatSafe conducted additional qualitative in-depth interviews and targeted behavioral research to complement the findings of this study and further inform intervention decisions [11,48,49].

Although it was not the primary focus of this study, it is key to highlight the role of market infrastructure in designing effective interventions. Respondents in this study reported several infrastructure issues that can contribute to the safety of foods sold at the market, including drainage and flooding, mud, improper waste disposal, and lack of potable water. Infrastructure has been described as a key gap to improving food safety in market settings by several other studies [41,50] and should be addressed along with behavior change and its drivers.

## 5. Conclusions

The results of this study highlight that vendors and consumers in traditional markets are motivated to sell and buy safe food products. As such, there are opportunities to increase food safety through the behaviors of consumers and vendors handling and purchasing food. The acceptability of food safety cues and the relative importance of other decision factors may direct or limit actions consistent with increased safety. The findings of this study could be practically applied in several ways, including the following:

- Interventions may leverage existing motivations (e.g., consumer demand for quality, or preference for certain shops) to direct or to nudge behaviors towards safer practices while being cognizant of trade-offs (e.g., price vs. quality/safety);
- Practices that currently promote food safety could be reinforced or modified to increase their effectiveness (e.g., keeping food elevated from the ground), while unhelpful practices could be discouraged;
- Existing risk perceptions and beliefs, as well as cues used to assess if a food is safe, could be reinforced when supporting food safety and corrected or deemphasized if not;
- Whether vendors’ awareness of what consumers look for matches actual purchase choice drivers of consumers can determine whether different messages are needed for consumers and vendors;
- External obstacles to carrying out a behavior (e.g., lack of clean water or inability to keep tools at the market overnight) could be addressed by an in-market intervention, as well as by advocacy for infrastructure or policy changes;
- Understanding competition and collaboration dynamics among vendors can direct behavior change strategies (e.g., using food safety as individual competitive advantage versus working in peer groups to uphold higher standards);
- Knowing what media sources individuals use can help select the most effective vehicles for messaging and interactive initiatives;
- Knowing what gender primarily conducts what activities can help determine whether different interventions should be focused towards women and/or men.
- The survey tools tested in this study may be customized and used by other researchers working in food markets or in other food environments.

Drivers and leverage points for behavior change investigated in this study could be further explored during intervention design and tested during intervention evaluation, as was done in the EatSafe program [32,51]. When key information on practices, motivations, and market operations needs to be obtained with minimal time and resources, novel tools such as the EatSafe rapid market assessment [52,53] can be particularly helpful. Customizing food safety behavior change interventions to local motivations, existing practices, and the enabling environments can increase the relevance of interventions for participants and support the inclusion of new behaviors into established mental models and routines.

## Figures and Tables

**Table 1:** Consumer and vendor demographics.

Characteristic	Category	Demographics	
		Vendors (N = 150)	Consumers (N = 150)
		**Mean (SD)**	**Mean (SD)**
Number of household residents		5.1 (2)	4.7 (2.1)
Number of household residents <5 years of age		0.6 (0.8)	0.5 (0.7)
Age (years)		30.5 (11)	32 (10)
		**N (%)**	**N (%)**
Sex	Men	22 (15%)	52 (35%)
	Women	128 (85%)	98 (65%)
Marital Status	Married	109 (73%)	94 (63%)
	Not married	32 (21%)	47 (31%)
Education ^1^	Divorced	3 (2%)	4 (3%)
	Widowed	6 (4%)	5 (3%)
	Primary (0–4th Grade)	32 (21%)	12 (8%)
	Secondary (5–12th Grade)	94 (63%)	78 (52%)
	Post-secondary	5 (3%)	44 (29%)
	Post-secondary (tvet) ^2^	4 (3%)	9 (6%)
	Never attended school (illiterate)	12 (8%)	3 (2%)
Language ^3^	Amharic	87 (58%)	118 (79%)
	Sidama	6 (4%)	12 (8%)
	Wolayita	55 (37%)	16 (11%)

1: Note: Seven respondents are excluded from the table and described here instead. Two respondents (N = 1 each consumer and vendor) indicated they only had kindergarten, nursery, or pre-school education. One consumer indicated they had informal education (i.e., they can read and write but have never attended any school). Four respondents (N = 2 each consumer and vendors) had non-regular education (e.g., adult literacy program, satellite schooling, or religious education). 2: TVET refers to technical and vocational education and training. 3: Six respondents’ primary language were Kenbatigna (N = 1 each consumer and vendor), Guragegna (N = 1 each consumer and vendor), Oromifa (N = 1 consumer), and sign language (N = 1 consumer).

**Table 2:** Consumers’ purchasing behaviors.

	Responses		Responses, by Gender	
Foods ^1^	N	% ^1^	Men N (%) ^2^	Women N (%) ^2^
Tomatoes	139	93%	49 (35%)	90 (65%)
Leafy greens	126	84%	38 (30%)	88 (70%)
Roots/tubers	80	53%	26 (33%)	54 (68%)
Legumes	66	44%	19 (29%)	47 (71%)
Eggs	25	17%	7 (28%)	18 (72%)
Poultry	21	14%	6 (29%)	15 (71%)
Grains	17	11%	1 (6%)	16 (94%)
Milk or Dairy Products	9	6%	0 (0%)	9 (6%)

1: Fish was not purchased at the market by any of the individuals surveyed, as there is a separate fish market in the city. 2: Total percentages reflect the full sample size (N = 150). Percentages by gender reflect the sample (N) per commodity, which varied, as respondents could skip questions or provide multiple answers per question.

**Table 3:** Consumer perceptions related to food safety.

Perception	Agreement				
	Strongly Disagree	Disagree	Neither Agree nor Disagree	Agree	Strongly Agree
People get sick from eating kale	19%	55%	10%	15%	1%
People get sick from eating lettuce	15%	61%	11%	13%	1%
People get sick from eating tomatoes	15%	47%	13%	23%	2%
Food safety differs between vendors	3%	10%	11%	65%	10%
Trust that vendors sell safe food	3%	15%	10%	63%	9%
Prefer to buy from vendors that have a food safety certification or license (if available)	6%	28%	5%	49%	13%

**Table 4:** Consumers’ trusted sources of information on food safety.

Media	Responses		Responses, by Gender	
	N	% ^1^	Men, N (%) ^2^	Women, N (%) ^2^
Medical professional (doctor/nurse)	100	67%	34 (34%)	66 (66%)
Friends or family	94	63%	35 (37%)	59 (63%)
Food packaging/labels	71	47%	27 (38%)	44 (62%)
Experts on the radio or TV	50	33%	22 (44%)	28 (56%)
Internet/social media	44	29%	16 (36%)	28 (64%)
Journalists (newspaper)/show hosts (TV/radio)	24	16%	12 (50%)	12 (50%)
Local religious leader	15	10%	6 (40%)	9 (60%)
A famous person you like	9	6%	6 (67%)	3 (33%)
Government agencies	4	3%	1 (25%)	3 (75%)

1: Total percentages reflect the full sample size (N = 150). 2: Percentages by gender reflect the sample (N) per commodity, which varied, as respondents could skip questions or provide multiple answers per question.

**Table 5:** Vendor choices of suppliers related to food safety (N = 150).

Perception	Agreement				
	Strongly Disagree % (N)	Disagree % (N)	Neither % (N)	Agree % (N)	Strongly Agree % (N)
Can find suppliers that sell high-quality foods	2% (3)	4% (6)	3% (4)	66% (99)	25% (38)
Knowing how to choose safe foods	1% (2)	1% (1)	1% (1)	65% (97)	33% (49)
Will spend a bit more time selecting safer foods	1% (1)	3% (4)	4% (6)	62% (93)	31% (46)
Will spend a bit more money selecting safer foods	0% (0)	2% (3)	3% (5)	62% (93)	33% (49)

**Table 6:** Vendor satisfaction with different aspects of operations in their shop (N = 150).

Perception	Agreement				
	Strongly Disagree % (N)	Disagree % (N)	Neither ^1^ % (N)	Agree % (N)	Strongly Agree % (N)
Proud of the quality of the food sold	2% (3)	1% (1)	1% (1)	58% (87)	39% (58)
Satisfied with shop operations	3% (4)	10% (15)	8% (12)	52% (78)	27% (41)
Rules for preserving food quality/safety exist	15% (23)	21% (31)	10% (15)	45% (67)	9% (14)
Rules for keeping the shop clean exist	14% (21)	23% (34)	7% (11)	45% (68)	11% (16)
It is sometimes difficult to keep the shop clean	13% (19)	35% (53)	3% (4)	39% (58)	11% (16)

^1^ “Neither agree nor disagree” was the middle category in the agreement scale.

## Data Availability

Data, data dictionaries, and survey questionnaires are provided as Supplemental Information.

## Supplementary Materials

The following supporting information can be downloaded at: https://www.mdpi.com/article/10.3390/ijerph22111645/s1. Supplemental Information S1: Consumer questionnaire. Supplemental Information S2: Vendor questionnaire. Supplemental Information S3: Ethiopia-specific PPI indicators and scores. Supplemental Information S4: De-identified datasets.

## References

[1] Havelaar A.H., Kirk M.D., Torgerson P.R., Gibb H.J., Hald T., Lake R.J., Praet N., Bellinger D.C., de Silva N.R., Gargouri N. (2015). World Health Organization Global Estimates and Regional Comparisons of the Burden of Foodborne Disease in 2010. PLOS Med..

[2] WHO (2015). WHO Estimates of the Global Burden of Foodborne Diseases.

[3] Jaffee S., Henson S., Unnevehr L., Grace D., Cassou E. (2018). The Safe Food Imperative: Accelerating Progress in Low-and Middle-Income Countries.

[4] Global Alliance for Improved Nutrition (GAIN) (2022). Review of Food Safety Policy and Legislation in Ethiopia. EatSafe: Evidence and Action Towards Safe, Nutritious Food. https://www.gainhealth.org/sites/default/files/publications/documents/Review%20of%20Food%20Safety%20Policy%20in%20Ethiopia.pdf.

[5] Grace D. (2015). Food Safety in Low and Middle Income Countries. Int. J. Environ. Res. Public. Health.

[6] Ayalew H. (2013). Review on food safety system: Ethiopian perspective. Afr. J. Food Sci..

[7] Roesel K., Grace D. (2014). Food Safety and Informal Markets: Animal Products in Sub-Saharan Africa.

[8] Nordhagen S., Hagos S., Gebremedhin G., Lee J. (2025). Vendor capacity and incentives to supply safer food: A perspective from urban Ethiopia. Food Secur..

[9] Grace D., Dipeolu M., Alonso S. (2019). Improving food safety in the informal sector: Nine years later. Infect. Ecol. Epidemiol..

[10] Parikh P., Aparo N.O., Nordhagen S., De Steur H. (2022). Food safety-related perspectives and practices of consumers and vendors in Ethiopia: A scoping review. Food Res. Int..

[11] Nordhagen S., Hagos S., Gebremedhin G., Lee J. (2024). Understanding consumer beliefs and choices related to food safety: A qualitative study in urban Ethiopia. Public Health Nutr..

[12] Isanovic S., Constantinides S.V., Frongillo E.A., Bhandari S., Samin S., Kenney E., Wertheim-Heck S., Nordhagen S., Holdsworth M., Dominguez-Salas P. (2023). How Perspectives on Food Safety of Vendors and Consumers Translate into Food-Choice Behaviors in 6 African and Asian Countries. Curr. Dev. Nutr..

[13] Lazaro J., Kapute F., Holm R.H. (2019). Food safety policies and practices in public spaces: The urban water, sanitation, and hygiene environment for fresh fish sold from individual vendors in Mzuzu, Malawi. Food Sci. Nutr..

[14] Global Alliance for Improved Nutrition (GAIN) (2022). Qualitative Behavioral Research in Traditional Markets in Kebbi State, Nigeria. EatSafe: Evidence and Action Towards Safe, Nutritious Food. https://www.gainhealth.org/resources/reports-and-publications/qualitative-behavioral-research-traditional-food-markets-kebbi.

[15] Bass S.B., Brajuha J., Kelly P.J., D’Avanzo P., Lambertini E., Nordhagen S., Monterrosa E.C. (2022). Changing Behavior, Attitudes, and Beliefs About Food Safety: A Scoping Review of Interventions Across the World and Implications for Empowering Consumers. Foodborne Pathog. Dis..

[16] USAID Advancing Nutrition (2021). Methods, Tools, and Metrics for Evaluating Market Food Environments in Low-and Middle-Income Countries.

[17] Turner C., Kalamatianou S., Drewnowski A., Kulkarni B., Kinra S., Kadiyala S. (2020). Food Environment Research in Low- and Middle-Income Countries: A Systematic Scoping Review. Adv. Nutr..

[18] Global Alliance for Improved Nutrition (GAIN) (2023). EatSafe Learnings from Phase I Research in Hawassa, Ethiopia. EatSafe: Evidence and Action Towards Safe, Nutritious Food. https://www.gainhealth.org/sites/default/files/publications/documents/EatSafe-Learnings-from-Phase-I-Research-in-Ethiopia.pdf.

[19] Gazu L., Alonso S., Mutua F., Roesel K., Lindahl J.F., Amenu K., Maximiano Sousa F., Ulrich P., Guadu T., Dione M. (2022). Foodborne Disease Hazards and Burden in Ethiopia: A Systematic Literature Review, 1990–2019. https://cgspace.cgiar.org/handle/10568/120999.

[20] Global Alliance for Improved Nutrition (GAIN) (2022). Literature Review on Foodborne Disease Hazards in Food and Beverages in Ethiopia. EatSafe: Evidence and Action Towards Safe, Nutritious Food. https://www.gainhealth.org/sites/default/files/publications/documents/Literature%20Review%20on%20Foodborne%20Disease%20Hazards%20in%20Foods%20and%20Beverages%20in%20Ethiopia.pdf.

[21] Global Alliance for Improved Nutrition (GAIN) (2023). Food Safety Hazards and Risk Associated with Fresh Vegetables: Assessment from a Traditional Market in Southern Ethiopia. https://www.gainhealth.org/sites/default/files/publications/documents/Food_Safety_Hazards_Risk_Fresh_Vegetables_%20Traditional_Market_Southern_Ethiopia.pdf.

[22] Hsieh F.Y., Liu A.A. (1990). Adequacy of sample size in health studies. Stanley Lemeshow, David W. Hosmer Jr., Janelle Klar and Stephen K. Lwanga published on behalf of WHO by Wiley, Chichester, 1990. No. of pages: xii + 233. Price:£D17.50. Stat. Med..

[23] StataCorp (2021). Stata Statistical Software: Release 17.

[24] The R Core Team (2022). R: A Language and Environment for Statistical Computing.

[25] Poverty Probability Index (2020). Ethiopia. In: PPI [Internet]. https://www.povertyindex.org/country/ethiopia.

[26] Misra S., Li H., He J. (2020). Machine Learning for Subsurface Characterization.

[27] Osafo R., Balali G.I., Amissah-Reynolds P.K., Gyapong F., Addy R., Nyarko A.A., Wiafe P. (2022). Microbial and Parasitic Contamination of Vegetables in Developing Countries and Their Food Safety Guidelines. J. Food Qual..

[28] Jaffee S., Henson S. (2024). Promoting Food Safety in the Informal Markets of Low and Middle-Income Countries: The Need for a Rethink. Food Prot. Trends.

[29] Rheinländer T., Olsen M., Bakang J.A., Takyi H., Konradsen F., Samuelsen H. (2008). Keeping up appearances: Perceptions of street food safety in urban Kumasi, Ghana. J. Urban Health.

[30] Nordhagen S., Lee J., Onuigbo-Chatta N., Okoruwa A., Monterrosa E., Lambertini E., Pelto G.H. (2022). “Sometimes You Get Good Ones, and Sometimes You Get Not-so-Good Ones”: Vendors’ and Consumers’ Strategies to Identify and Mitigate Food Safety Risks in Urban Nigeria. Foods.

[31] Lee J., Pelto G.H., Nordhagen S. (2022). Beliefs, values, and sociocultural patterns related to food safety in low- and middle-income countries: A synthesis of the descriptive ethnographic literature. Appetite.

[32] Global Alliance for Improved Nutrition (GAIN) Leveraging Consumer Demand to Drive Food Safety Improvements in Traditional Markets: FTF EatSafe’s Research & Implementation Results. https://www.gainhealth.org/resources/reports-and-publications/leveraging-consumer-demand-drive-food-safety-improvements.

[33] Noor A.Y.M., Toiba H., Setiawan B., Wahib Muhaimin A., Nurjannah N. (2024). Indonesian Consumers’ Preferences and Willingness to Pay for Certified Vegetables: A Choice-Based Conjoint Approach. J. Int. Food Agribus. Mark..

[34] Hoffmann V., Moser C.M., Herrman T.J. (2021). Demand for Aflatoxin-Safe Maize in Kenya: Dynamic Response to Price and Advertising. Am. J. Agric. Econ..

[35] Owusu-Sekyere E., Owusu V., Jordaan H. (2014). Consumer preferences and willingness to pay for beef food safety assurance labels in the Kumasi Metropolis and Sunyani Municipality of Ghana. Food Control.

[36] Alimi B.A., Oyeyinka A.T., Olohungbebe L.O. (2016). Socio-economic characteristics and willingness of consumers to pay for the safety of *fura de nunu* in Ilorin, Nigeria. Qual. Assur. Saf. Crops Foods.

[37] Wongprawmas R., Canavari M. (2017). Consumers’ willingness-to-pay for food safety labels in an emerging market: The case of fresh produce in Thailand. Food Policy.

[38] Alphonce R., Alfnes F. (2012). Consumer willingness to pay for food safety in Tanzania: An incentive-aligned conjoint analysis. Int. J. Consum. Stud..

[39] Alimi B.A., Workneh T.S. (2016). Consumer awareness and willingness to pay for safety of street foods in developing countries: A review. Int. J. Consum. Stud..

[40] Vuong H., Pannell D., Schilizzi S., Burton M. (2024). Vietnamese consumers’ willingness to pay for improved food safety for vegetables and pork. Aust. J. Agric. Resour. Econ..

[41] Zhong S., Werner C. (2025). The hidden strength of small business: Social networks and wet market vendors in China. Econ. Anthropol..

[42] Nordhagen S., Lee J., Monterrosa E., Onuigbo-Chatta N., Okoruwa A., Lambertini E., Pelto G.H. (2023). Where supply and demand meet: How consumer and vendor interactions create a market, a Nigerian example. Food Secur..

[43] Nordhagen S., Onuigbo-Chatta N., Lambertini E., Wenndt A., Okoruwa A. (2023). Perspectives on food safety across traditional market supply chains in Nigeria. Food Humanit..

[44] Wenndt A., Nordhagen S., Okoruwa A., Onuigbo-Chatta N., Swartz H., Andohol P., Igho S., Lambertini E. (2025). Food safety through the eyes of rural market vendors in northwest Nigeria. J. Rural. Stud..

[45] Hennessey M., Kim S., Unger F., Nguyen-Viet H., Dang-Xuan S., Nguyen-Thi T., Häsler B. (2020). Exploring the potential of using nudges to promote food hygiene in the pork value chain in Vietnam. Prev. Vet. Med..

[46] Wallace F., Mittal N., Lambertini E., Nordhagen S. (2022). Vendor Knowledge, Attitudes, and Practices Related to Food Safety in Low- and Middle-Income Countries: A Scoping Review. J. Food Prot..

[47] Global Alliance for Improved Nutrition (GAIN) (2022). Food Safety Attitudes and Practices in Traditional Markets in Nigeria: A Quantitative Formative Assessment. https://www.gainhealth.org/resources/reports-and-publications/food-safety-attitudes-and-practices-traditional-markets-nigeria.

[48] Global Alliance for Improved Nutrition (GAIN) Food Safety Perceptions and Practices in Ethiopia: A Focused Ethnographic Study. https://www.gainhealth.org/resources/reports-and-publications/food-safety-perceptions-and-practices-ethiopia-focused.

[49] Global Alliance for Improved Nutrition (GAIN) (2022). Evaluation of Consumer and Vendor Behaviors in a Traditional Food Market in Hawassa, Ethiopia. EatSafe: Evidence and Action Towards Safe, Nutritious Food. https://www.gainhealth.org/sites/default/files/publications/documents/Evaluation%20of%20Consumer%20and%20Vendor%20Behaviors%20in%20a%20Traditional%20Food%20Market%20in%20Hawassa%2C%20Ethiopia.pdf.

[50] Ghatak S., Srinivas K., Milton A.A.P., Priya G.B., Das S., Lindahl J.F. (2023). Limiting the spillover of zoonotic pathogens from traditional food markets in developing countries and a new market design for risk-proofing. Epidemiol. Health.

[51] Global Alliance for Improved Nutrition (GAIN) (2024). Leveraging Consumer Demand to Drive Food Safety Improvements in Traditional Markets—An Activity Implementation Guide. https://www.gainhealth.org/sites/default/files/publications/documents/intervention-implementation-guide.pdf.

[52] Global Alliance for Improved Nutrition (GAIN) (2024). Rapid Market Assessment Tool for Food Safety in Traditional Markets. https://www.gainhealth.org/resources/reports-and-publications/rapid-market-assessment-tool-food-safety-traditional-markets.

[53] Global Alliance for Improved Nutrition (GAIN) (2024). Market Assessment Tools for Traditional Markets. https://www.gainhealth.org/resources/reports-and-publications/market-assessment-tools-traditional-markets.

