# Supplementary material for: Food Safety Practices and Behavior Drivers in Traditional Food Markets in Ethiopia: Assessing the Potential for Consumer-Driven Interventions
Source: Int J Environ Res Public Health. 2025 Oct 29;22(11):1645. doi: 10.3390/ijerph22111645 (PMC12652902; doi:10.3390/ijerph22111645)
Supplement: Supplementary file 1 [file ijerph-22-01645-s001.zip › S1_Consumer_Survey_Instrument.pdf]

Food Safety Practices and Behavior Drivers in Traditional Food Markets in Ethiopia: assessing the potential for consumer-driven interventions

Ariel V. Garsow, Smret Hagos, Eric Djimeu, Carrel Fokou, Haley Swartz, Genet Gebremedhin, Bisaku Chacha, and Elisabetta Lambertini

5.1 Supplemental Information 1: Consumer Survey

GAIN EatSafe Ethiopia Formative Research
Consumer Survey Instrument

INTERVIEWER NOTE

For the purposes of the survey, some definitions and abbreviations include:

- Key Commodities: the general commodities that are the focus of the study (see Table 1)
- Key Foods: specific forms or preparations (recipes) made with the Key Commodities
- "Foods": generic term used in the survey to include both commodities or specific food forms, when the distinction is not important.
- FS: food safety

A List of the Key Commodities and associated Key Foods, mentioned in the survey, are shown in Table 1 and Table 2. The list of Key Foods will be further refined during the survey pilot.

Table 1. List of the Categories and associated Key Commodities considered in the survey.

Key Commodity Category	Key Commodities
	Raw/fresh
Fresh vegetables	Kale (Gomen)
Fresh vegetables	Lettuce
Fresh vegetables	Tomatoes

Table 2. List of the specific Key Foods

Key Commodity Category	Key Foods
	Raw/fresh
Fresh vegetables	Kale (Gomen)
Fresh vegetables	Lettuce
Fresh vegetables	Tomatoes

None of the answers should be read out loud, unless there are explicit directions to do this in a specific question. Pre-coded answers are provided to facilitate recording answers. What the pre-coded answer options are should be assessed and refined during testing. Unless otherwise specified, answers include an "Other (specify)" category to be filled by the verbatim answer as needed. All pre-coded response options are single unless otherwise stated. All interviewer instructions are in bold text and should not be readout to the respondent.

MODULE 1.1: Consent and Survey information

INTERVIEWER NOTE: **Check that the respondent is a knowledgeable member of the household and at least 18 years old before proceeding. You may not interview a child under the age of 18.**

INTERVIWER READOUT: Good morning / Good evening Madam / Sir, my name is [name of interviewer], and I work for SART on behalf of IPSOS and GAIN, SART is a research organisation here in Hawassa. We are conducting this survey to better understand households' experiences and needs when shopping for food , in particular at traditional markets in this area. You were enrolled because you regularly buy food at Aroge Gebeya market. The survey will take approximately 45 minutes in total. We know that your time is precious. To show our appreciation for your help, we offer a thank-you gift for those that complete the survey. All the information we collect will remain strictly confidential, and results will only be shared in anonymous form and summarized over a group. This survey is carried out for the Global Alliance for Improved Nutrition (GAIN), an international non-profit organization that works on food and nutrition. While we have permission from the municipal authorities to conduct this survey, individual information you share will not be seen by any local organization or individual.

Also, no response or responses provided will be associated with any individual in line with Ipsos Global Privacy and Data Protection Policy and the Ethiopia Data Protection Regulations 2019 (EDPR). For more information, please read our Privacy Policy on Ipsos_Global_Privacy_and_Data_Protection_Policy.pdf at www.ipsos.com or contact us on +251 911 53 44 06

Participating in this interview is completely voluntary. If you do not wish to answer a question or wish to stop the interview, please let me know. You are free to withdraw without any penalty or loss of benefits to which you are otherwise entitled. We hope you can participate in this survey since your opinions are very important. The first part of the interview will be about characteristics of your household. The rest of the survey will ask about experiences related to buying food, with focus on some specific food categories and characteristics.

S0.0 Do you have any questions? **[INTERVIEWER WAIT FOR RESPONSE AND ANSWER WITHIN THE CONTEXT OF THE SURVEY]**

S0.1 Do you agree to start with the interview?
1. Yes [Proceed to Q1.1.1]
2. No [Auto-code 5 at Q1.1.2 and proceed to the end of the script - Salutation]

Q#	Question	Answer
1.1.1	Date	Day/month/year
1.1.2	Survey status<br><br>1.1.2= 1: Continue<br>1.1.2= 2 or 3: Appointment Scheduling and proceed to the end of the script.<br>1.1.2= any of codes 4 to 9, proceed to the end of the script.	1. Consented/available for interview<br>2. No HH member at home<br>3. Postponed/request new appointment<br>4. Entire HH absent for extended period<br>5. Refused<br>6. Dwelling vacant or not a dwelling<br>7. Dwelling destroyed<br>8. Dwelling not found

		9. Other (specify)
1.1.3	Interviewer ID	
1.1.4	Supervisor	Name
1.1.5	Interview start time	hr:min (in 24 hr notation)
1.1.6	Select Market where consumer was enrolled<br><br>[Note for scripter: this field can be pre-filled]	1 Aroge Gebeya
1.1.7	Language of interview	1 Amharic<br>2 Sidama<br>3 Welayta<br>4 Other (specify)
1.1.8	Sub city and Kebele	Text and number
1.1.8a	Village	
1.1.9	Enter the House number	Number
1.1.10	Capture and show GPS coordinates/ GPS<br>[lock in GPS]	XX:XX:XX

**MODULE 1.2: Demographics**		
**INTERVIEWER SAY**: This first set of question is about the demographics of your household.		
**Q#**	**Question**	**Answer**
	Time Stamp	HH:MM:SS
1.2.1	First Name of Respondent	[open-ended]
1.2.2	Last Name of Respondent	[open-ended]
1.2.3	Gender of respondent (Observed, not asked)	1 Male<br>2 Female
1.2.4	How old are you, in completed years?	Numeric
1.2.5	What is your marital statuws?	1. Not married<br>2. Married<br>3. Divorced<br>4. Widowed

		77 Other (specify)
1.2.6	Are you the head of the household?	1 Yes<br>2 No
1.2.7	[If 1.2.6 = "No"]<br><br>What is your relationship to the head of the household?	1 Spouse (primary)<br>2 Spouse (one of several)<br>3 Child<br>4 Child In-Law<br>5 Parent<br>6 Parent In-Law<br>7 Sibling<br>8 Aunt/Uncle<br>9 Nephew/Niece<br>10 Grandparent<br>11 Sibling In-Law<br>12 Domestic Help or Related to Domestic Help<br>13 Other Relative of HH<br>14 Not Related to HH<br>77. Other (specify)<br>99 Don't Know
1.2.8	What is the highest level of school you have completed?	1 Kindergarden, Nursery, Pre-School<br>2 0 – 4th Grade<br>3 5th Grade – End of Secondary<br>4 Post-Secondary (College or University)<br>5 Post-Secondary (TVET)<br>6 Informal Education (can read and write but has never attended any school)<br>7 Non-Regular Education (e.g. Adult Literacy Program, Satellite, or Religious Education)<br>8 Never attended school and cannot read and write<br>77 Other (specify; manually reassign to category)<br>99 Don't Know/Not Sure
1.2.9	[If 1.2.6_pre = "No"]<br><br>What is the highest level of school that the head of household has completed?	1 Kindergarden, Nursery, Pre-School<br>2 0 – 4th Grade<br>3 5th Grade – End of Secondary<br>4 Post-Secondary (College or University)<br>5 Post-Secondary (TVET)<br>6 Informal Education (can read and write but has never attended any school)<br>7 Non-Regular Education (e.g. Adult Literacy Program, Satellite, or Religious Education<br>8 Never attended school and cannot read and write<br>77. Other (specify; manually reassign to category)<br>99. Don't Know/Not Sure
1.2.10	What is your main occupation?	1. Self-employed in the food sector (production, harvest, supply chain, retail/vending, catering, restaurant)

		2. Weekly/daily wage labor in the food sector<br>3. Self-employed-non-food sector<br>4. Weekly/daily wage labor-non-food sector<br>5. Salaried worker<br>6. Occasional or seasonal work<br>7. Student<br>8. Unpaid worker/volunteer<br>9. Does not work / Unemployed<br>10. Retired<br>77. Other (specify)
1.2.12	How many people currently live in your household, defined as people that eat meals at the house?	Number [Allow only numbers]
1.2.13	[If 1.2.12 >1] How many household members are under age 5 years?<br>[1.2.12 cannot be greater than or equal to 1.2.11]	Number [Allow only numbers]
1.2.14	[If 1.2.12 >1] How many household members are between 5 and 18 years of age?<br>[1.2.12 + 1.2.13 cannot be greater than or equal to 1.2.11]	Number [Allow only numbers]
1.2.15	Over the past one week (7 days), did you or others in your household consume any beef?	1 Yes<br>0 No
1.2.16	Over the past one week (7 days), did you or others in your household consume any horse beans?	1 Yes<br>0 No
1.2.17	What language is most commonly used in your household?	1 Amharic<br>2 Sidama<br>3 Wolayita<br>77. Other (specify)
1.2.18	For how many years have you lived in Hawassa? **(years. If <1 year, record as "0" category)**	Number [Allow only numbers]

**MODULE 1.3: Household characteristics and assets**		
**INTERVIEWER SAY**: I will now ask you some quick questions on your house and items your household may own		
**Q#**	**Question**	**Answer**

1.3.1	Did your household have access to electricity in the past 12 months?	1 yes<br>0 no
1.3.2	What is the main source of water used by the household? **(select one)**	1 Piped water (to dwelling, plot, or neighboring plot)<br>2 Private tap, well, or standpipe<br>3 Public tap, well, or standpipe<br>4 Spring<br>5 Rainwater<br>6 Surface water (river, dam, lake, pond, stream, etc.)<br>7 Tanker truck<br>8 Packaged water (Plastic jar, bottles, sachets, etc.)<br>99. Don't Know<br>77. Other (specify)
1.3.3	What is the main construction material of the roof of the dwelling? **Answered by observation (not asked to respondent) if interview is conducted in the home**	A. Thatch<br>B. Mud and Wood<br>C. Bamboo/Reed<br>D. Plastic Canvas<br>E. Corrugated Iron Sheets<br>F. Concrete/Cement<br>G. Asbestos<br>H. Bricks<br>77. Other (specify)
1.3.4	How many separate rooms do the members of the household occupy? Do not count bathrooms, toilets, storerooms, or garage?	Number [Allow only numbers]
1.3.5	What is the main source of light for the household?	A. Biogas<br>B. Electrical battery<br>C. Light from dry cell with switch<br>D. Kerosene light lamp (imported)<br>E. Local kerosene lamp (Kuraz)<br>F. Candle/Wax<br>G. Firewood<br>H. Electricity meter-private<br>I. Electricity meter-shared<br>J. Electricity from generator<br>K. Solar energy<br>L. Lantern<br>77. Other (specify)
1.3.6	What is the main source of cooking fuel for the household?	A. Firewood<br>B. Crop residue/leaves

		C. Dung/Manure<br>D. Saw dust<br>E. Solar energy<br>F. Biogas<br>G. Purchased firewood<br>H. Charcoal<br>I. Kerosene<br>J. Butane-Gas<br>K. Electricity<br>M. None<br>77. Other (specify)
1.3.7	What kind of toilet facility does the household use?	A. PIT Latrine without slab<br>B. Composting toilet<br>C. Field/Forest<br>D. Flush toilet<br>E. PIT Latrine (ventilated PIT)<br>F. PIT Latrine with slab<br>G. Bucket<br>77. Other
**INTERVIEWER SAY**: Now I'm going to ask if you or your household owns any of the following items.		
1.3.8	Does your household own a television?	1 Yes<br>0 No
1.3.8a	Does your household have satellite dish?	1 Yes<br>0 No
1.3.9	Does your household own a radio?	1 Yes<br>0 No
1.3.10	How many mobile phones does the household own?	Number [Allow only numbers]
1.3.11	Does anyone currently residing in the household own a smart phone?	1 Yes<br>0 No
1.3.12	[If 1.3.10 > 0] Which type(s) of mobile phone do you own, personally, if any? (select all that apply)	1 Mobile<br>2 Smart<br>3 None<br>77 Other (specify)
1.3.13	[If 1.3.10 > 0 or 1.3.11=1 AND if marital status is MARRIED] Which type(s) of mobile phone does your spouse own, personally, if any? (select all that apply)	1 Mobile<br>2 Smart<br>3 None<br>77Other (specify)

1.3.14	[If 1.3.10 = 0 or 1.3.11=2] Even if you do not own a phone, do you have access to one when you need it?	1 Yes<br>0 No
1.3.15	Which motorized vehicles, if any, does your household own? (select all that apply)	1 Car<br>2 Truck<br>3 Motorbike<br>4 TVS tricycle<br>5 None<br>77 Other (specify)
1.3.16	Does anyone currently residing in the household own a Computer	1 Yes<br>0 No
1.3.17	Does your household own a Refrigerator	1 Yes<br>0 No
1.3.18	Does your household cultivate any food crops?	1 Yes<br>0 No
1.3.19	[if 1.3.18 = "Yes"]<br><br>What food crops does your household cultivate?	1 Grains<br>2 Pulses<br>3 Starchy Tubers and Roots<br>4 Vegetables<br>5 Fruits<br>77 Other (specify)
1.3.20	Does any member of your household own any land? This includes both agricultural and non-agricultural land.	1 Yes<br>0 No
1.3.21	Does this household own any livestock, herds, other farm animals, or poultry?	1 Yes<br>0 No
1.3.22	How many mattresses does your household own?	Number

**MODULE 1.4: Household shopping habits and choices**		
**INTERVIEWER SAY:** Next we would like to ask you about how your households acquires or consumes food.		
**Q#**	**Question/ Tambaya**	**Answer/ Amsa**
1.4.1	Are you the member of the household who is primarily responsible for going to buy the food that is prepared at home?<br>(If one of several primary shoppers, select "Yes")	1 Yes<br>0 No
1.4.2	[If 1.2.12 >1] Who else in the household is responsible for going to	1 Spouse<br>2 Children

	buy the food that is prepared at home?	3 Other Relative (living in household)<br>4 Other Relatives (not living in household)<br>5. Someone from another household helps me<br>6. House maid<br>7. I am the only responsible person<br>77 Other (specify)<br>99. Don't Know
1.4.3	[If 1.2.12Are you a primary decision-maker about food purchases for your household?<br><br>(If one of several primary decision-makers, select "Yes")	1 Yes<br>0 No
1.4.4	[If 1.2.12 >1] Who else in the household decides which foods to purchase, whether or not they go to the market to buy the foods? List all that apply.<br><br>NOTE TO INTERVIEWER: Selected relations should be with respect to the respondent, not the head of household	1 My spouse<br>2 My children<br>3 My parents (one or both)<br>4 Other Relative (living in household)<br>5 Other Relatives (not living in household)<br>6. Someone from another household who helps me<br>77 Other (specify)<br>99 Don't Know
1.4.5	Approximately what percent of your household's food do you purchase, from any kind of market, store, or seller, rather than by self-production or other non-purchase means?	[Should not allow more than 100%]
1.4.6	Where do you regularly purchase food for your household? This question refers to the food that is prepared and eaten at home, as opposed to a ready-to-eat meal or snack you might have while away from the house. Name all that apply.	1 Individual shop not in a market or mall<br>2 Local/traditional market<br>3 Wholesale market<br>4 Supermarket<br>5 Street vendors<br>77 Other (specify)
1.4.7 (loop)	[LOOP for each source noted above] how frequently do you buy food from there, on average?	1 Daily or almost<br>2 Weekly (at least once a week or more)<br>3 Monthly (at least once a month or more)<br>4 Every 2-3 months<br>5 Occasionally (around 2-3 times/year or less)<br>77 Other (specify)
1.4.8	Are you a primary food preparer for the household?	1. Yes<br>2. No<br>3. Unsure

	(If one of several primary food preparers, select "Yes")	
1.4.9	[If 1.2.12 >1] Who else prepares and cooks food in the household?	1 My spouse<br>2 My children<br>3 My parents (one or both)<br>4 Other Relative (living in household)<br>5 Other Relatives (not living in household)<br>6.House made<br>77 Other (specify)
1.4.10	How often does the household eat ready-to-eat foods, i.e. meals that are not prepared at home, not including small snacks? (eaten both at home and elsewhere)	1 Daily or almost<br>2 Weekly (at least once a week or more)<br>3 Monthly (at least once a month or more)<br>4 Every 2-3 months<br>5 Occasionally (around 2-3 times/year or less)<br>6 Never or almost<br>77 Other (specify)
1.4.11	When buying food for your HH, what attributes or characteristics of a food are most important to you? List up to four.<br><br>[Scripter: ensure answers appear in the data in the same order they are selected – Allow only 4 answers]	1 Freshness<br>2 Price<br>3 Safety<br>4 Nutritional content orHealthiness<br>5 Appearance (size, shape, color)<br>6 Ease of preparation<br>7 Taste<br>8 Appropriateness for recipes<br>9 Household member satisfaction<br>10 Balanced or varied diet<br>11 Food appears clean and free from dirt<br><br>77 Other (specify)
1.4.12	[If 1.2.12 > 0]<br>On a scale of 1 – 5, where 1=Never, 2= Rarely, 3=Sometimes, 4=Often and 5=Always, How often do children <5 years old in your HH eat the same food prepared for the rest of the family?	1 Never [skip 1.4.14]<br>2 Occasionally/Rarely [skip 1.4.14]<br>3 Sometimes<br>4 Often<br>5 Every time or almost
1.4.13	[If 1.2.12 > 0]<br>When buying food for your young children, what food attributes or characteristics do you look for? List up to four.<br><br>[Scripter: ensure answers appear in the data in the same order they are selected – Allow up to 4]	1 Freshness<br>2 Price<br>3 Safety<br>4 Nutritional content orHealthiness<br>5 Appearance (size, shape, color)<br>6 Ease of preparation<br>7 Taste<br>8 Appropriateness for recipes<br>9 Household member satisfaction<br>10 Balanced or varied diet<br>11 Food appears clean and free from dirt

		77 Other (specify)
1.4.14	On a scale of 1 – 5, where 1= Not at all satisfied and 5= Completely satisfied, how satisfied are you with the available options for places to buy foods from?	1 Not at all satisfied<br>2 Slightly dissatisfied<br>3 Neither satisfied nor dissatisfied<br>4 slightly satisfied<br>5 Completely satisfied
1.4.15	On the same scale from 1 to 5, how satisfied are you with how healthy the foods your household eats are?	1 Not at all satisfied<br>2 Slightly dissatisfied<br>3 Neither satisfied nor dissatisfied<br>4 slightly satisfied<br>5 Completely satisfied

MODULE 2: Consumer practices and behaviors in markets

**MODULE 2.1: Choices and behaviors related to the study market**		
**INTERVIEWER SAY:** I am now going to ask you about your habits and experiences shopping at the [Pipe-in name of market from 1.1.6] market. This second section will take approximately 15 minutes or less.		
	Time stamp	hr:min
**Q#**	**Question**	**Answer**
2.1.1	Is [pipe-in response at 1.1.6] the market you most frequently buy food at?	1 Yes<br>0 No
2.1.2	How many other local/traditional markets do you regularly visit to buy food?	Number
2.1.3	How frequently do you shop at [pipe-in response at 1.1.6] market?	1 Daily or almost<br>2 Weekly (at least once a week or more)<br>3 Monthly (at least once a month or more)<br>4 Every 2-3 months<br>5 Occasionally (around 2-3 times/year or less)<br>77 Other (specify)
2.1.4	What are the top 3 reasons you shop for food at [pipe-in response at 1.1.6], as opposed to some othr market?<br><br>**Note for interviewer: record answers in the order they are given. Do not ask the respondent to rank their answers. Allow only three answers**.<br><br>[Scripter: ensure answers appear in the data in the same order they are selected]	1 Proximity or it is easy to get to<br>2 Food Quality<br>3 Food Prices<br>4 Market Cleanliness<br>5 Food Safety or Cleanliness<br>6 Food Variety<br>7 Vendor Characteristics<br>77 Other (specify)
2.1.5	For how many years or months have you been shopping for food at this market?	1 Less than two months<br>2 Between two and 12 months<br>3 Between one and three years<br>4 More than three years<br>99. Don't Know
2.1.6	Which types of foods do you usually buy at [Market Name]?	1 Leafy greens (e.g. cabbage, lettuce, kale)<br>2 Tomatoes<br>3 Grains or flours<br>4 Legumes<br>5 Roots or tubers<br>6 poultry (e.g., chicken)<br>7 Fresh fish<br>8 Eggs

		9 Milk or dairy<br>77. Other (specify)
2.1.7	What time of day do you usually shop for food at this market?	1. 06:00 – 08:00<br>2. 08:00 – 12:00<br>3. 12:00 – 14:00<br>4. 14:00 – 17:00<br>5. After 17:00<br>6. No "usual" time<br>77. Other (specify)
2.1.8	In general, how secure do you feel while shopping at this market, on a scale of 1 – 5, from 1=Very insecure to 5=Very secure.<br>Secure is intended here as physical or personal safety, i.e. safe from being harassed, or from crimes such as theft or assault.<br>[Interviewer: if asked, explain the scale: 1=Very insecure, 2= Somewhat insecure, 3=Moderately, 4=Somewhat secure and 5=Very secure.]	1 Very insecure<br>2 Somewhat insecure<br>3 Neutral<br>4 Somewhat secure<br>5 Very secure
2.1.9	Thinking of your last shopping experience at [pipe-in response at 1.1.6], on a scale from 1=very dirty to 5=very clean, how would you rate [pipe-in response at 1.1.6] in terms of cleanliness?<br><br>[Interviewer: If asked, explain scale: 1= very dirty, 2=dirty, 3 = not too clean, but not too dirty either, 4 = clean, 5 = very clean]	1 Very dirty<br>2 Dirty<br>3 Not too clean, but not too dirty either<br>4 Clean<br>5 Very clean
2.1.10	[If 2.1.4 is not = 4] Is a market's cleanliness ever a reason for choosing which market you buy from?	1 Yes<br>0 No
2.1.11	[If 2.1.9=1/2/3] What makes this market dirty?	[Open-Ended]

2.1.12	On a scale of 1 – 5 where 1= Not at all satisfied to 5= Completely satisfied, considering the last year, overall, how satisfied are you with your shopping experience at [pipe-in response at 1.1.6]?<br><br>[Interviewer: if asked, explain the scale: 1= Not at all satisfied, 2= Slightly satisfied, 3= Neither satisfied nor dissatisfied<br>4 slightly satisfied, and 5= Completely satisfied]	1 Not at all satisfied<br>2 Slightly disatisfied<br>3 Neither satisfied nor dissatisfied<br>4 slightly satisfied<br>5 Completely satisfied
2.1.13	[If 2.1.12 = 1 or 2 or 3] For what reasons, if any, has your shopping experience been less than satisfactory at this market in the past?	[Open-ended]

**MODULE 2.2: Choices of and interactions with VENDORS/SHOPS**		
**INTERVIEWER SAY:** These next questions refer to your choices of which shops or vendors to buy food from.		
**Q#**	**Question/Tambaya**	**Answer/Amsa**
2.2.1	At [pipe-in response at 1.1.6], during a typical food shopping trip, approximately how many shops do you acquire food from? Please include stalls, kiosks, and transient vendors in and around the market.	Number [Allow only numbers]
2.2.2	On a scale of 1 – 5, where 1=Never, 2= Rarely, 3=Sometimes, 4=Often and 5=Always,<br>when you shop at [pipe-in response at 1.1.6], how often do you compare shops/stalls before deciding where to buy food from?	1 Never<br>2 Occasionally/rarely<br>3 Sometimes<br>4 Often<br>5 Every time or almost
2.2.3	[If answer to 2.2.2 is 3, 4 or 5] For which foods do you compare shops at least sometimes?	[see list in 2.1.6]
2.2.4	[If answer to 2.2.2 is 3, 4 or 5] On average, for [commodity mentioned in 2.2.3], how many shops do you look at or compare, before deciding where to get that commodity?<br><br>[SCRIPTER: if possible, loop so this is asked for each commodity mentioned in 2.2.3]	Number
2.2.5	When you get food at the Aroge Gebeya [Market name], do you feel you have plenty of	1. Usually have plenty of time<br>2. Usually in a hurry

	time to look for the best food, or are you often in a hurry?	3. Sometimes I have time, sometime not<br>77. Other (specify)
2.2.6	On a typical shopping visit, approximately how much time do you spend at the market?	Number (minutes)
2.2.6b	When you go to the market, do you only buy food, or do you also engage in other social activities? Select all that apply	1 I only buy food [if selected, exclude other answers]<br>2 I talk to friends or people I know<br>3 I converse with vendors, beyond just the transactions<br>4 I talk to other customers I may not know<br>5 I listen to town criers or other public announcements<br>5 I get information I need<br>6 I watch people<br>77. Other (specify)
2.2.7	What method do you use, if any, to remind yourself of which food to buy at the market? Name all that apply.	1. I carry a written shopping list<br>2. I know in my head what I need<br>3. I decide what to buy depending on what I see at the market.<br>77. Other (Specify)
2.2.8	What characteristics of a shop or vendor would make you want to purchase from them again in the future? List up to 3 most important characteristics.<br><br>**Note for interviewer: record answers in the order they are given. Do not ask the respondent to rank their answers. Allow only three answers**.<br><br>[Scripter: ensure answers appear in the data in the same order they are selected – Allow only three]	1 Prices<br>2 Food quality (other than cleanliness/ safety)<br>3 Food cleanliness/safety<br>4 Food Variety<br>5 Credit<br>7 Possibility to barter<br>8 Vendor personality/how they treat me as their customer<br>9 Comfort/Security<br>10 Trust in vendor<br>11 Personal/Family Connection<br>12 Consistent food availability<br>13 Additional services they offer (specify)<br>77-Other (specify)
2.2.9	Considering the vendors you shop from during a typical visit to [pipe-in response at 1.1.6], approximately what percentage of these vendors would you consider yourself a “regular customer” of, meaning you shop from them very often or nearly every time you visit the market to buy the type of food they sell?	% [0-100, Should not allow more than 100%]
2.2.10	Specifically, when you buy fresh vegetables such as tomatoes or kale at [pipe-in response at 1.1.6], on a scale from 0 to 100% (never to every	% [0-100, Should not allow more than 100%]<br>e

	time) how often do you buy them from a vendor you are a regular customer of?	
2.2.11	[If answer to 2.2.10 is >0%] Compared to other vendors or shops, what do your regular vendors do or have that makes you prefer them to others? [Select all that apply and include verbatim any answer not in pre-scripted options; as always, do not read answers aloud]	1. Prices<br>2. Food quality (other than cleanliness/ safety)<br>3. Food cleanliness/safety<br>4. Food Variety<br>5. Credit<br>6. Possibility to barter<br>7. Vendor personality/how they treat me as their customer<br>8. Comfort/Security<br>9. Trust in vendor<br>10. Personal/Family Connection<br>11. Consistent foodavailability<br>12. Additional services they offer (specify)<br>77-Other (specify)
2.2.12	What specific characteristics would you look for in a shop, if you wanted to assess its cleanliness or hygiene? Name all that apply.	1 Orderliness/Organization of shop<br>2 Surfaces are present (e.g. counter, table, board)<br>3 Surfaces appear free from fluids or debris<br>4 Food is not on the ground/floor<br>5 Food is covered/contained<br>6 Cover/container cleanliness<br>7 Waste management<br>8 Pest/animal presence<br>77 Other (specify)
2.2.13	When considering the hygiene behavior of a vendor in managing their shop, what would you look for in what the vendor does or does not? Name all that apply.	[Open-ended LIST]
2.2.13b	When acquiring food at the market, approximately for what percentage of your purchases do you barter, instead of paying with money?	% [0-100]
**INTERVIEWER SAY:** These next questions are about specific actions related to shopping and choosing food at the [pipe-in response at 1.1.6]. They all refer to the timeframe of the last year, that is approximately from June 2021 to now (July 2022).		

2.2.14	In the last year, how often have you brough a complaint to a vendor about the food they sell, or the food they had sold you?	1 Daily or almost<br>2 Weekly (at least once a week or more)<br>3 Monthly (at least once a month or more)<br>4 Every 2-3 months<br>5 Occasionally (around 2-3 times/year or less)<br>6 Never<br>77 Other (specify)
2.2.15	[If answer to 2.2.14 is not "Never"]<br><br>What were the characteristics of food that you most often complained to vendors about?<br>[Allow up to 3 answers]	1. Quality<br>2. Shelf Life/food did not keep well<br>3. Food made customer or HH member sick<br>4. Taste<br>5. Price<br>6. Household satisfaction<br>7. Error in type or amount of food<br>77. Other (specify)
2.2.16	For fresh vegetables specifically, how often have you brough a complaint to a vendor about the food they sell, or the food they had sold you?	1 Daily or almost<br>2 Weekly (at least once a week or more)<br>3 Monthly (at least once a month or more)<br>4 Every 2-3 months<br>5 Occasionally (around 2-3 times/year or less)<br>6 Never<br>77 Other (specify)
2.2.17	[If answer to 2.2.16 is not "Never"] What were the characteristics of the vegetables that you complained to vendors about?<br>[Allow up to 3 answers]	1. Type of vegetable<br>2. Size or shape of the vegetables<br>3. Blemishes<br>4. Shelf Life/food did not keep well<br>5. Food made customer or HH member sick<br>6. Taste<br>7. Price<br>8. Household satisfaction<br>9. Error in type or amount of food<br>77. Other (specify)
2.2.18	On a scale of 1 to 5 (from 1=not at all necessary to 5=very necessary], to what extent do people in your community think that when buying food, it is necessary to check a shop's hygiene or cleanliness?	1. Not at all necessary<br>2. Not necessary<br>3. Neutral<br>4. Necessary<br>5. Very necessary
2.2.19	Also, on a scale of 1 to 5, to what extent do people in your community think that when buying food, it is necessary to check the vendor's personal hygiene?	1. Not at all necessary<br>2. Not necessary<br>3. Neutral<br>4. Necessary<br>5. Very necessary

2.2.20	How often – if at all - have you decided to stop buying from a specific vendor for any reason, in the past year?	1 Never [Skip to 2.2.22]<br>2 Occasionally/Rarely<br>3 Sometimes<br>4 Often<br>5 Every time or almost Very Often
2.2.21	What were the reasons, if any, that you have stopped buying food from a specific vendor in the past? List up to three reasons.<br><br>**Note for interviewer: record answers in the order they are given. Do not ask the respondent to rank their answers. Allow only three answers**.<br><br>[Scripter: ensure answers appear in the data in the same order they are selected – Allow only three]	1 Prices<br>2 Food quality (other than cleanliness/ safety)<br>3 Food Variety<br>4 Reasons related to credit<br>5 Shop cleanliness/hygiene<br>6 Shop location/not easy to get to<br>7 Shop appearance<br>8 Vendor personalityrelationship//how they treat me as their customer<br>9 Reasons related to trust or breach of trust<br>10 omfort/Security issues<br>6. Personal/family connection reasons<br>7. Bartering not available<br>8. Long waiting time, or long line<br>77 Other (specify)
2.2.22	How often have you had conversations with food vendors about aspects of food safety in the past year?	1. Never [Skip to 2.2.26]<br>2. Rarely<br>3. Sometimes<br>4. Often<br>5. Every time or almost
2.2.23	How likely are you to start (i.e. be the initiator of) a conversation with a vendor about any aspects of the safety of foods?	1 Very unlikely<br>2 Unlikely<br>3 Neutral<br>4 Likely<br>5 Very Likely
2.2.24	[if 2.2.22 is not = 1] In conversations with vendors about food safety, what specific foods do you most often talk about?	[see list in 2.1.6]

2.2.25	What topic(s) related to the quality of food did you most often discuss, in these conversations with vendors?	[Open-ended]
2.2.26	Can you name some actions that, in your opinion, vendors at [pipe-in response at 1.1.6] can take to improve the safety of the food they sell?	OPEN-END

MODULE 2.3: Behaviors related to choosing FOODS at the market

INTERVIEWER SAY: The next questions refer to what features of the food itself impact your decision to buy it, when shopping at [pipe-in response at 1.1.6] for food that will be prepared at home

Q#	Question	Answer
2.3.1	You told us what food characteristics are important to you. Thinking specifically of whether a food is safe or unsafe to eat, how would you define "safety"? (name all that apply)<br><br>[Scripter: separate multiple answers from the same responder with a ";"]	[Open-ended]

INTERVIEWER SAY: Here in this survey, when we talk about "food safety" we will mean that a food, when you buy it, does not contain harmful chemicals or germs that can make people ill, e.g. with stomach upset, poisoning, or cancer.

2.3.2	From your perspective, what do you think are signs that a batch of food might not be safe? List all the signs that come to mind.	[Open-ended list]
2.3.3	Thinking of vegetables you purchase fresh, what are the signs that a batch of vegetables might not be safe?	[Open-ended list]
2.3.4	What do you or the main food preparer in your HH do, if anything, after you bring [pipe-in list of commodities] home from the market, to make sure that it will be safe when eaten?<br><br>[Select from Key Commodities listed in Table 1]	1- Nothing [if selected, exclude other answer options]<br>2 Washing<br>3 Heating (cooking, boiling, etc.)<br>5-Eat it promptly<br>6-Dried it<br>7 Preserved it (e.g. pickled, fermented, salted)<br>77-Other (specify)
2.3.5	Assume that you wanted to buy a kilogram of tomatoes, and their price in this market is 30 Birr, and someone offers to sell tomatoes to you for 25 Birr, but you felt that these are less safe to eat, would you buy them?	1. Yes<br>2. No<br>3. Unsure

2.3.6	Why did you answer [pipe in answer from 2.3.5]?	[Open-ended LIST]

MODULE 3: Consumer perceptions, beliefs, and attitudes

**MODULE 3.1: Beliefs and attitudes related to VENDORS/SHOPS and MARKET**		
**INTERVIEWER SAY: This third section focuses more on your opinions and personal perspectives, and it will take approximately 10 minutes or less.** These next questions are about how you view vendors and shops at the [Market name].		
**Q#**	**Question**	**Answer**
**Beliefs/Fahimta**		
I'm going to read you a few statements, some more general and some about vendors at [pipe-in response at 1.1.6] and would like to know whether you agree or disagree with the statement, based on your own perspective, on a 1-5 scale from 1-Strongly disagree; 2-Disagree; 3-Neither agree nor disagree; 4-Agree; 5-Strongly agree. For example, if the statement is "I like to eat fish," my personal response might be "strongly agree" if I really like eating fish, or my response might be "neither agree nor disagree" if I sometimes eat fish but don't like it a lot, and so on. The scale is the same for all questions, so I won't repeat it. [Interviewer: do not explain the scale for each question; only repeat the scale if the respondent is confused or asks.]		
3.1.1	"It is common that people get sick from eating [Pipe in food type]"<br><br>[Ask for each Key Food]	1 Strongly disagree<br>2 Disagree<br>3 Neither agree nor disagree<br>4 Agree<br>5 Strongly agree
3.1.2	"At [Market name], some vendors sell food that is safer to eat than other vendors."<br>~~READ OUT RATING SCALE~~	1 Strongly disagree<br>2 Disagree<br>3 Neither agree nor disagree<br>4 Agree<br>5 Strongly agree
3.1.3	"Some vendors at [pipe-in response at 1.1.6] care more about food safety than others."	1 Strongly disagree<br>2 Disagree<br>3 Neither agree nor disagree<br>4 Agree<br>5 Strongly agree
3.1.4	"I trust that the vendors I like to buy from at [pipe-in response at 1.1.6] sell me food that is safe."	1 Strongly disagree<br>2 Disagree<br>3 Neither agree nor disagree<br>4 Agree<br>5 Strongly agree
3.1.5	I would prefer to buy food from a vendor that displays some sort of food safety certification or license, if one was available, compared to a vendor who does not.	1 Strongly disagree<br>2 Disagree<br>3 Neither agree nor disagree<br>4 Agree<br>5 Strongly agree
3.1.6	"If I had the option to buy the same food from a male vendor or a female vendor, I would buy from the female."	1 Strongly disagree<br>2 Disagree<br>3 Neither agree nor disagree<br>4 Agree<br>5 Strongly agree

3.1.7	"In general, female vendors are more trustworthy than male vendors."	1 Strongly disagree<br>2 Disagree<br>3 Neither agree nor disagree<br>4 Agree<br>5 Strongly agree
3.1.8	"In general, female vendors sell better quality of food than male vendors."	1 Strongly disagree<br>2 Disagree<br>3 Neither agree nor disagree<br>4 Agree<br>5 Strongly agree
3.1.9	"In general, female vendors take more care to keep their food clean and protected than male vendors."	1 Strongly disagree<br>2 Disagree<br>3 Neither agree nor disagree<br>4 Agree<br>5 Strongly agree
3.1.10	"I trust that what vendors at [pipe-in response at 1.1.6] tell me about the food they sell is accurate."	1 Strongly disagree<br>2 Disagree<br>3 Neither agree nor disagree<br>4 Agree<br>5 Strongly agree
3.1.11	"I trust that vendors at [pipe-in response at 1.1.6] will sell me food at fair prices."	1 Strongly disagree<br>2 Disagree<br>3 Neither agree nor disagree<br>4 Agree<br>5 Strongly agree
3.1.12	In general, who do you think should be primarily responsible for ensuring that the food that is sold at the market is safe? Name up to 4<br><br>[Scripter: ensure answers appear in the data in the same order they are selected – Allow up to four selections]	[Open-ended]
3.1.13	[If code 1 or 2 in 3.1.3] You mentioned that you aren't always confident that there are options at this market for buying safe food. What is the reason(s) for this?<br><br>[Scripter: ensure answers appear in the data in the same order they are selected]	[Open-ended]
**ATTITUDES: WHAT A CONSUMER VALUES KNOWING ABOUT A SHOP**		
**INTERVIEWER SAY:** The next questions will ask you to rate certain characteristics on a scale of how important they are to you, from 1="Very Low Importance" to 5="Very High Importance"		
3.1.14	How important is it to you that you know how food is handled in a shop?	1 Very Low Importance<br>2 Low Importance<br>3 Moderate Importance

	~~READ OUT RATING SCALE~~	4 High Importance<br>5 Very High Importance
3.1.15	How important is it to you that you know where the food you buy comes from?	1 Very Low Importance<br>2 Low Importance<br>3 Moderate Importance<br>4 High Importance<br>5 Very High Importance
**[Attitudes: How vendors act around and interact with customers]**		
3.1.16	How important is it to you that you can talk to a vendor about questions and concerns related to the food they sell?	1 Very Low Importance<br>2 Low Importance<br>3 Moderate Importance<br>4 High Importance<br>5 Very High Importance
3.1.17	How important is it to you that you can see a vendor actively taking care of their shop?	1 Very Low Importance<br>2 Low Importance<br>3 Moderate Importance<br>4 High Importance<br>5 Very High Importance
3.1.18	How important is it to you that a vendor has good personal hygiene?	1 Very Low Importance<br>2 Low Importance<br>3 Moderate Importance<br>4 High Importance<br>5 Very High Importance
3.1.19	How often do you talk to other customers to share advice regarding food purchasing decisions?	1 Never<br>2 occasionally/Rarely<br>3 Sometimes<br>4 Often<br>5 Every time or almost

**MODULE 3.2: Perceptions on FOOD characteristics**		
**Q#**	**Question**	**Answer**
3.2.1	How satisfied are you overall about the food you buy at [pipe-in response at 1.1.6]?<br><br>**READ OUT RATING SCALE**	1 Very Unsatisfied<br>2 Unsatisfied<br>3 Neither Satisfied nor Unsatisfied<br>4 Satisfied<br>5 Very Satisfied
3.2.2	What factors related to the food at [pipe-in response at 1.1.6] are you most satisfied with? List up to three most satisfying factors.	1 Quality<br>2 Variety<br>3 Price<br>4 Safety<br>5 Healthiness<br>6 Cleanliness<br>7 I know where the food is from

	[Scripter: ensure answers appear in the data in the same order they are selected, three selections required]	8 Quantity<br>9 Household satisfaction<br>10 None<br>10 77 Other (specify)
3.2.3	What factors related to the food at [pipe-in response at 1.1.6] are you least satisfied with? List upto three least satisfying factors.	1. Quality<br>2. Variety<br>3. Price<br>4. Safety<br>5. Healthiness<br>6. Cleanliness<br>7. I know where the food is from<br>8. Quantity<br>9. Household satisfaction<br>10. None<br>77.Other (specify)
3.2.4	Which foods in [pipe-in response at 1.1.6] if any, are you least likely to be satisfied with?	[Select from Key Commodities listed in Table 1]<br>1. None

**MODULE 3.3: Health concerns**		
The next short series of questions, which concludes this section, is about health concerns.		
**Q#**	**Question**	**Answer**
3.3.1	Which food-borne diseases or issues, if any, are you most worried about for your household? Name up to three.	[Open-ended]
LOOP the following two questions (3.3.2 and 3.3.3) for each food-borne disease specified in 3.3.1:		
3.3.2	[For each concern selected in 3.3.1] Which food(s) are associated with this food-borne disease or issue? Name upto three.	[Select from Key Foods listed in Table 2]
3.3.3	[For each food selected in 3.3.2] Why do you think [food selected in 3.3.2] is associated with [foodborne illness selected in 3.3.1]?	1. Microbial contamination<br>2. Chemical contamination<br>3. Dirt/Filth<br>4. Processing methods<br>5. Preparation methods<br>6. Lack of hygiene<br>7. Other (specify)
END LOOP		

3.3.4	Have you or someone in your HH experienced serious sickness related to consuming a particular food in the past year? By "seriously sick", we mean that it was difficult or impossible to work or perform daily activities.	1 Yes<br>0 No<br>Don't Know/Not Sure
3.3.5	Have you or someone in your HH experienced serious sickness related to drinking water in the past year?	1 Yes<br>0 No<br>Don't Know/Not Sure

MODULE 4: Media use and sources of information

MODULE 4.1: Sources of information		
Module Start Time: HH:MM		
**INTERVIEWER SAY**: We are three quarters of the way through the survey. The next questions relate to what sources of information you access, and your use of media		
**Q#**	**Question**	**Answer**
4.1.1	Who do you trust to provide reliable information about health issues? (select all that apply)	1. Medical professional (doctor nurse)<br>2. Community member<br>3. Community health worker<br>4. Family member<br>5. Religious leader<br>6. Community leader<br>7. News paper<br>8. Experts on radio or TV<br>77. Other (specify)
4.1.2	Suppose you wanted to find out if the food you eat is safe. Would you consult [source] for information?<br><br>Yes/No for each source, asked aloud	1. Friends or family<br>2. Medical professionals, such as your local doctor or nurse.<br>3. Experts on Newspapers, television, or radio;<br>4. Journalists/show hosts on newspapers, television, or radio<br>5. The internet/social media<br>6. Bureau of Trade and Industry/Health<br>7. The packaging or label on the food<br>8. A famous person you like<br>9. Local religious leaders<br>77. Other (specify)
4.1.3	Can you name a specific issue regarding food that you wanted to know more	1 Yes<br>2 None<br><br>4.1.3.is 1 (If yes,)<br>Open-ended

	about, or a question you had, in the last year?	
4.1.3b	If Q 4.1.3 is yes	Open ended
4.1.4	[If 4.1.3=1] Specifically, was there anything about food being safe or unsafe, that you wanted to know more about?	1 Yes<br>0 No<br>4.1.4.if 1 (If yes), what was the topic you were interested in? Open-ended
4.1.4b	If Q4.1.4 is yes	Open enfded
4.1.5	[If 4.1.4=1] If you looked for that information, which resources or people did you lseek out?<br>(Select all that apply)	1. Friends or family<br>2. Medical professionals, such as your local doctor or nurse.<br>3. Experts on Newspapers, television, or radio; Journalists/show hosts on Newspapers, television, or radio the internet/social media<br>4. Bureau of Trade and Industry/Health<br>5. The packaging or label on the food<br>6. A famous person you like<br>7. Local religious leaders<br>77. Other (specify)<br>88. Did not look for information

MODULE 4.2: Media usage		
**Q#**	**Question**	**Answer**
Module Start Time: HH:MM		
4.2.1	How often do you use the internet, including social media, per week (for any use)?	1 Never → **GO TO 4.2.6**<br>2 a few times per week<br>3 most days but not every day<br>4 every day
4.2.2	On what device(s) do you usually access Internet? List all applicable devices.	1 Smartphone<br>2 Mobile Tablet<br>3 Laptop Computer<br>4 Desktop Computer<br>77 Other (specify)
4.2.3	[For each selection in 4.2.2]<br><br>Do you personally own one or more of this type of device?	1 Yes<br>0 No
4.2.4	Where do you usually access internet, for personal use? List all applicable places.	1 Home<br>2 On my phone, wherever I am<br>3 Friend’s, Relative's, or Neighbor’s Home<br>4 Internet Café<br>5 Public Library<br>77 Other (specify)

4.2.5	What social media platform do you regularly use, if any? Select all that applyM	1 Facebook<br>2 Twitter<br>3 YouTube<br>4 WhatsApp<br>5 Instagram<br>6 Telegram<br>7 Tik Tok<br>8 None<br>77 Other (specify)
4.2.6	Which media channels do you use for entertainment? By entertainment we mean content or activities that bring amusement, enjoyment, or relaxation (as opposed to obtaining information or carryin gout a practical task).<br><br>*Select the top four channels you use.*	1 Normal TV/<br>2 Satellite TV<br>3 Radio<br>4 Movie theatre/cinema<br>5 Internet<br>6 Social media<br>7 Messaging apps<br>77 Other (specify)
4.2.7	For each of the channels selected, how often do you use them?<br>[Pipe-in selection from 4.2.6 and apply response as drop-down per selection]	1-daily<br>2- 2 or 3 times per week<br>3-once a week<br>4-once every two weeks<br>5-once per month
4.2.8	When you are looking for entertainment, what content do you most often seek out?<br><br>(select all that apply)	1. TV films/series/soap operas<br>2. TV talk shows<br>3. Radio serials or fictional stories<br>4. Radio talk shows<br>5. Online videos<br>6. Chats on social media<br>7. Meeting in person<br>77. Others (specify)
Module End Time: HH:MM		

**MODULE 5: Food acquisition recall**		
**INTERVIEWER SAY:** We are almost done. This last set of questions, which will take approximately 5 minutes, is about the types and amounts of foods that your household consumed and acquired in the past week. We will ask the same questions for Three foods, focusing on how much of each food you use when preparing a typical meal for your whole household.		
**Q#**	**Question**	**Answer**
5.1	Did your household participate in any festival or celebration in the last 7 days, that made last week atypical in terms of foods bought or consumed?	1 Yes<br>0 No
5.2	In the past week (7 days), how many times (meals) did your household consume foods made from ... ?<br><br>**READ KEY FOOD LIST. [Multiple responses possible]**<br><br>**NOTE FOR INTERVIEWER:** If food was not consumed in past 7 days, enter "0"	[Loop: ask separately for each Key Foods, i.e. kales, tomatoes, lettuce]
5.6	What percentage of the [Key Commodity X] consumed by your household in the past 7 days came from PURCHASES?	Percent (for each commodity)
5.13	Does the amount of [Key Food X] consumed in your household vary season-to-season?	1 Yes<br>0 No
5.14	[If Yes to 5.13] Please indicate the season(s) in which [Key Food X] is consumed the most, in your household. Select all that apply	Select season(s)<br><br>1 Kiremt or Meher (Summer) - June, July and August<br>2 Belg (Autumn) - September, October and November<br>3 Bega (Winter) - December, January and February<br>4 Tseday (Spring) - March, April and May<br>77 Other period (pecify)
5.15	[If Yes to 5.13] In [SEASON WHEN Key Food X IS CONSUMED THE MOST], how would you compare the amount consumed by your HH in a typical week of that [season/month], compared to the amount you reported for the last 7 days?	1. Much Less<br>2. Somewhat Less<br>3. About the Same<br>4. Somewhat More<br>5. Much More
5.16	Does the amount of [Key Food X] that comes from PURCHASES vary season-to-season?	1 Yes<br>0 No

5.17	[If yes to 5.16] Please indicate the seasons in which [Key Food X] is purchased the most by your household (as opposed to self-produced or acquired by other means)	Select season(s)
5.18	[If yes to 5.16] In [SEASON WHEN Key Food X IS PURCHASED], how would you rate the amount you purchase in a typical week of that season, compared to the level you reported for the last 7 days?	1. Much Less<br>2. Somewhat Less<br>3. About the Same<br>4. Somewhat More<br>5. Much More

[Survey Closing]: We are at the end for the survey. Thank you very much for sharing your valuable opinion. The information received from you and from other participants will be very useful in making food safer in wet markets. To express our appreciation, we would like to give you a thank you token of _____birr for you time you spend with us. iThis study will conduct other surveys in the next two years. Could we contact you again at that time?
1. Yes
2. No
Thank you again [Salutation]

1.1.5b	Interview end time	hr:min (in 24 hr notation)
