# Supplementary material for: Food Safety Practices and Behavior Drivers in Traditional Food Markets in Ethiopia: Assessing the Potential for Consumer-Driven Interventions
Source: Int J Environ Res Public Health. 2025 Oct 29;22(11):1645. doi: 10.3390/ijerph22111645 (PMC12652902; doi:10.3390/ijerph22111645)
Supplement: Supplementary file 1 [file ijerph-22-01645-s001.zip › S2_Vendor_Survey_Instrument.pdf]

Food Safety Practices and Behavior Drivers in Traditional Food Markets in Ethiopia: assessing the potential for consumer-driven interventions

Ariel V. Garsow, Smret Hagos, Eric Djimeu, Carrel Fokou, Haley Swartz, Genet Gebremedhin, Bisaku Chacha, and Elisabetta Lambertini

5.2 Supplemental Information 2: Vendor Survey

GAIN EatSafe Ethiopia Formative Research
Vendor Survey Instrument

INTERVIEWER NOTE

For the purposes of the survey, some definitions and abbreviations include:
Revised

1. Key Commodities: the general commodities that are the focus of the study (see Table 1)
2. Key Foods: specific forms or preparations (recipes) made with the commodities listed above, e.g. bula made from maize, masa made from rice, maize flour.
3. "Foods": generic term used in the survey to include both commodities or specific food forms, when the distinction is not important.
4. FS: food safety

A List of the Key Commodities and associated Key Foods, mentioned in the survey, are shown in Table 1 and Table 2. The list of Key Foods will be further refined during the survey pilot.

Table 1. List of Key Commodities considered in the survey.

Key Commodity Category	Key Commodities
	Raw/fresh
Fresh vegetables	Kale (Gomen)
Fresh vegetables	Lettuce
Fresh vegetables	Tomatoes

Table 2. List of the specific Key Foods considered in the survey.

Key Commodity Category	Key Foods
	Raw/fresh
Fresh vegetables	Kale (Gomen)
Fresh vegetables	Lettuce
Fresh Vegetables	Tomato

None of the answers should be read out loud, unless there are explicit directions to do this in a specific question. Pre-coded answers are provided to facilitate recording answers. What the pre-coded answer options are should be assessed and refined during testing. Unless otherwise specified, answers include an "Other (specify)" category to be filled by the verbatim answer as needed. All pre-coded response options are single unless otherwise stated. All interviewer instructions are in bold text and should not be readout to the respondent.

MODULE 1.1: Consent and Survey information
**INTERVIEWER NOTE: Check that the respondent is a knowledgeable member of the household and at least 18 years old before proceeding. You may not interview a child under the age of 18.**

INTERVIWER READOUT: Good morning / Good evening Madam / Sir, my name is [name of interviewer], and I work for SART on behalf of IPSOS and GAIN, SART is , a research organisation here in Hawassa.We are conducting this survey to better understand your experience when selling food particular at this traditional market. You are enrolled because you regularly sell food at Aroge Gebeya market. . The survey will take approximately 45 minutes in total. We know that your time is precious. To show our appreciation for your help, we offer a thank-you gift for those that complete the survey. All the information we collect will remain strictly confidential, and results will only be shared in anonymous form and summarized over a group. This survey is carried out for the Global Alliance for Improved Nutrition, an international non-profit organization that works on food and nutrition. While we have permission from the municipal authorities to conduct this surve, individual information you share will not be seen by any local organization or individual.

Also, no response or responses provided will be associated with any individual in line with Ipsos Global Privacy and Data Protection Policy and the Ethiopia Data Protection Regulations 2019 (NDPR). For more information, please read our Privacy Policy on Ipsos_Global_Privacy_and_Data_Protection_Policy.pdf at www.ipsos.com or contact us on +251911534406
If you do not wish to answer a question or wish to stop the interview, please let me know. We hope you can participate in this survey since your opinions are very important. The first part of the interview will be about characteristics of your household. The rest of the survey will ask about experiences related to selling food, with focus on some specific food categories and characteristics.
S0.0 Do you have any questions? **[INTERVIEWER WAIT FOR RESPONSE AND ANSWER WITHIN THE CONTEXT OF THE SURVEY]**

S0.1 Do you agree to start with the interview?
1. Yes [Proceed to Q1.1.1]
2. No [Auto-code 5 at Q1.1.2 and proceed to the end of the script - Salutation]

Q#	Question	Answer
1.1.1	Date	Day/month/year
1.1.2	Survey status<br><br>1.1.2= 1: Continue<br>1.1.2= 2 or 3: Appointment Scheduling and proceed to the end of the script.<br>1.1.2= any of codes 4 to 9, proceed to the end of the script.	1. Consented/available for interview<br>2. No shopmember at shop/stall<br>3. Postponed/request new appointment<br>4. Entire shop/stall absent for extended period<br>5. Refused<br>6. Stall/Dwelling vacant or not a dwelling<br>7. Stall/Dwelling destroyed<br>8. Stall/Dwelling not found<br>77. Other (specify)
1.1.3	Interviewer ID	
1.1.4	Supervisor	Name
1.1.5	Interview start time	hr:min (in 24 hr notation)
1.1.6	Select Market where vendor was enrolled<br><br>[Note for scripter: this field can be pre-filled]	1 Aroge Gebeya

1.1.7	Language of interview/	1 Amharic<br>2 Sidama<br>3 Welayta<br>77. Other (specify)
1.1.8	Sub city and Kebele	Text and number
1.1.8a	Village	
1.1.10	Enter the house number	Number
1.1.11	Capture and show GPS coordinates/ GPS<br>[lock in GPS]	XX:XX:XX

**MODULE 1.2: Demographics**		
**INTERVIEWER SAY**: This first set of question is about the demographics of your household.		
**Q#**	**Question**	**Answer**
	Time Stamp	HH:MM:SS
1.2.1	First Name of Respondent	[open-ended]
1.2.2	Last Name of Respondent	[open-ended]
1.2.3	Gender of respondent (Observed, not asked)	1 Male<br>2 Female
1.2.4	How old are you, in completed years?	Numeric
1.2.5	What is your marital status?	1. Not married<br>2. Married<br>3. Divorced<br>4. Widowed<br>77. Other (specify)
1.2.6_pre	Are you the head of the household?	1 Yes<br>2 No
1.2.6	[If 1.2.6_pre = "No"]<br><br>What is your relationship to the head of the household?	1 Spouse (primary)<br>2 Spouse (one of several)<br>3 Child<br>4 Child In-Law<br>5 Parent<br>6 Parent In-Law<br>7 Sibling<br>8 Aunt/Uncle<br>9 Nephew/Niece<br>10 Grandparent<br>11 Sibling In-Law

		12 Domestic Help or Related to Domestic Help<br>13 Other Relative of HH<br>14 Not Related to HH<br>77. Other (specify)<br>99 Don't Know
1.2.7	What is the highest level of school you have completed?	1 Kindergarden, Nursery, Pre-School<br>2 0 – 4th Grade<br>3 5th Grade – End of Secondary<br>4Post-Secondary (College or University)<br>5 Post-Secondary (TVET)<br>6 Informal Education (can read and write but has never attended any school)<br>7 Non-Regular Education (e.g. Adult Literacy Program, Satellite, or Religious Education<br>8 Never attended school and cannot read and write<br>77 Other (specify; manually reassign to category)<br>99 Don't Know/Not Sure
1.2.8	[If 1.2.6 = "No"]<br><br>What is the highest level of school that the head of household has completed?	1 Kindergarden, Nursery, Pre-School<br>2 0 – 4th Grade<br>3 5th Grade – End of Secondary<br>4 Post-Secondary (College or University)<br>5 Post-Secondary (TVET)<br>6 Informal Education (can read and write but has never attended any school)<br>7 Non-Regular Education (e.g. Adult Literacy Program, Satellite, or Religious Education<br>8 Never attended school and cannot read and write<br>77 Other (specify; manually reassign to category)<br>99 Don't Know/Not Sure
1.2.11	How many people currently live in your household, defined as people that eat meals at the house?	Number [Allow only numbers]
1.2.12	[If 1.2.11 >1] How many household members are under age 5 years?<br>[1.2.12 cannot be greater than or equal to 1.2.11]	Number [Allow only numbers]
1.2.13	[If 1.2.9 >1] How many household members are between 5 and 18 years of age?<br>[1.2.12 + 1.2.13 cannot be greater than or equal to 1.2.11]	Number [Allow only numbers]

1.2.14	Over the past one week (7 days), did you or others in your household consume any beef?	1 Yes<br>0 No
1.2.15	Over the past one week (7 days), did you or others in your household consume any horse beans?	1 Yes<br>0 No
1.2.16	What language is most commonly used in your household?	1 Amharic<br>2 Sidama<br>3 Wolayita<br>77. Other (specify)
1.2.17	For how many years have you lived in Hawassa? **(years. If <1 year, record as "0" category)**	Number [Allow only numbers]

**MODULE 1.3: Household characteristics and assets**		
**INTERVIEWER SAY**: I will now ask you some quick questions on your house and items your household may own		
**Q#**	**Question**	**Answer**
1.3.1	Did your household have access to electricity in the past 12 months?	1 yes<br>0 no
1.3.2	What is the main source of water used by the household? **(select one)**	1 Piped water (to dwelling, plot, or neighboring plot)<br>2 Private tap, well, or standpipe<br>3 Public tap, well, or standpipe<br>4 Spring<br>5 Rainwater<br>6 Surface water (river, dam, lake, pond, stream, etc.)<br>7 Tanker truck<br>8 Packaged water (Plastic jar, bottles, sachets, etc.)<br>99 Don't Know<br>77 Other (specify)
1.3.4	What is the main construction material of the roof of the dwelling? **Answered by observation (not asked to respondent) if interview is conducted in the home**	A. Thatch<br>B. Mud and Wood<br>C. Bamboo/Reed<br>D. Plastic Canvas<br>E. Corrugated Iron Sheets<br>F. Concrete/Cement<br>G. Asbestos<br>H. Bricks<br>77 Other (specify)
1.3.5	How many separate rooms do the members of the household occupy?	Number [Allow only numbers]

	Do not count bathrooms, toilets, storerooms, or garage?	
1.3.6	What is the main source of light for the household?	A. Biogas
		B. Electrical battery
		C. Light from dry cell with switch
		D. Kerosene light lamp (imported)
		E. Local kerosene lamp (Kuraz)
		F. Candle/Wax
		G. Firewood
		H. Electricity meter-private
		I. Electricity meter-shared
		J. Electricity from generator
		K. Solar energy
		L. Lantern
		77. Other (specify)
1.3.7	What is the main source of cooking fuel for the household?	A. Firewood<br>B. Crop residue/leaves<br>C. Dung/Manure<br>D. Saw dust<br>E. Solar energy<br>F. Biogas<br>G. Purchased firewood<br>H. Charcoal<br>I. Kerosene<br>J. Butane-Gas<br>K. Electricity<br>M. None<br>77. Other (specify)
1.3.8	What kind of toilet facility does the household use?	A. PIT Latrine without slab<br>B. Composting toilet<br>C. Field/Forest<br>D. Flush toilet<br>E. PIT Latrine (ventilated PIT)<br>F. PIT Latrine with slab<br>G. Bucket<br>77. Other (specify)
**INTERVIEWER SAY**: Now I'm going to ask if you or your household owns any of the following items.		
1.3.9	Does your household own a television?	1 Yes<br>0 No

1.3.9a	Does your household have satellite dish	1 Yes<br>0 No
1.3.10	Does your household own a radio?	1 Yes<br>0 No
1.3.11	How many mobile phones does the household own?	Number [Allow only numbers]
1.3.12	Does anyone currently residing in the household own a smart phone?	1 Yes<br>0 No
1.3.13	[If 1.3.11 > 0] Which type(s) of mobile phone do you own, personally, if any? (select all that apply)	1 Mobile<br>2 Smart<br>3 None<br>77 Other (specify)
1.3.14	[If 1.3.11 > 0 or 1.3.12=1 AND if marital status is MARRIED] Which type(s) of mobile phone does your spouse own, personally, if any? (select all that apply)	1 Mobile<br>2 Smart<br>3 None<br>77 Other (specify)
1.3.15	[If 1.3.11 = 0 or 1.3.12=2] Even if you do not own a phone, do you have access to one when you need it?	1 Yes<br>0 No
1.3.16	Which motorized vehicles, if any, does your household own? (select all that apply)	1 Car<br>2 Truck<br>3 Motorbike<br>4 TVS tricycle<br>5 None<br>77 Other (specify)
1.3.17	Does anyone currently residing in the household own a computer	1 Yes<br>0 No
1.3.18	Does your household own a Refrigerator	1 Yes<br>0 No
1.3.19	Does your household cultivate any food crops?	1 Yes<br>0 No
1.3.20	[if 1.3.19 = “Yes”]<br><br>What food crops does your household cultivate? Select all that apply	1 Grains<br>2 Pulses<br>3 Starchy Tubers and Roots<br>4 Vegetables<br>5 Fruits<br>77 Other (specify)
1.3.23	Does any member of your household own any land? This includes both agricultural and non-agricultural land.	1 Yes<br>0 No
1.3.26	Does this household own any livestock, herds, other farm animals, or poultry?	1 Yes<br>0 No

1.3.22	How many mattresses does your household own?	Number

MODULE 1.4: Food business characteristics		
**INTERVIEWER SAY**: The next questions are about your work as a food vendor and how your food selling business at [pipe-in response at 1.1.6] operates.		
**Q#**	**Question**	**Answer**
1.4.1	Shop structure<br>**[Observed, not asked]**	1. stone/block/brick built structure<br>2. Wood structure<br>3. Table/stall without roof<br>4. Stall with plastic roof<br>5. Cart, baskets/containers<br>6. Tarp on floor<br>77. Other (specify)
1.4.2	How long have you been selling food at [pipe-in response at 1.1.6]?	Number of years (use fraction of a year for months)
1.4.3	Is the shop always in the same place, or do you move through the market?	1. Fixed and food is always in the same place<br>2. Fixed with some flexibility of where the food is displayed (e.g. additional tables/containers in front of the shop structure)<br>3. Moves but generally stays in the same area of the market; vendor walks around market<br>77. Other (specify)
1.4.4	How often in a typical week is the shop operating at [pipe-in response at 1.1.6]?	1. Every day<br>2. Every day except one day<br>3. 2-3 days/week<br>4. One day a week
1.4.8	Does the shop operate throughout the year, or for only part of the year?	1. All year<br>2. Seasonally
[If 1.4.8 = 2]<br><br>1.4.8_post	During which month(s) of the year is the shop operational? List all that apply.	1 Jan<br>2 Feb<br>3 Mar<br>4 Apr<br>5 May<br>6 Jun<br>7 Jul<br>8 Aug<br>9 Sep<br>10 Oct<br>11 Nov<br>12 Dec

1.4.9	**[Asked for Key Commodities. Capture one commodity per cell]**<br>What food products are regularly sold in the shop? (Select all that apply)	[Select from Key Commodities listed in Table 1]
1.4.11_pre	Does the type of food you sell vary with the time of the year?	1 Yes<br>0 No
[If 1.4.11_pre = 1]<br><br>1.4.11	If so, how?	[Open-ended]
1.4.13	[If more than 1 item listed in 1.4.9]<br>What foods bring in the most income for the shop?	[Pipe in entries from 1.4.9 code list. Allow up to three selections]
1.4.14	What is your primary role in the shop?	1. Owner<br>2. Main manager<br>3. Co-manager<br>4. Junior manager<br>5. Employee<br>6. Occasional helper<br>77. Other (specify)
1.4.17	Have you also worked in other shops that sell raw or fresh foods (that is, uncooked, non ready-to-eat) in the past?	1 Yes<br>0 No
1.4.18	Do you currently sell food any where else besides this market?	1 Yes<br>0 No
1.4.19	[If 1.4.18 = Yes]<br>In which types of markets or shops do you currently sell food, aside from this market?	1. Other traditional market<br>2. Village market (*gulit*)<br>3. Wholesale<br>4. Supermarket<br>5. Individual fixed shop/kiosk, not within a larger market<br>6. Individual mobile vendor such as using a cart or baskets, not within a larger market<br>77 Other (specify)
1.4.24	In the last year, have you done any other income-generating jobs in addition to	1 Yes (specify)<br>0 No

	working at this food shop? If yes, please specify.	
1.4.24a	How many people in total are employed by your shop at [1.1.6], including yourself?	Number [Allow only numbers]
1.4.24b	[If 1.4.24a>0] What percent of the employees that work at the shop are male?	% Male
1.4.25	In your shop at [pipe-in response at 1.1.6], how many people usually work at the shop at the same time?<br><br>NOTE FOR INTERVIEWER: If vendor works alone at the shop, enter "1"	Number [Allow only numbers]
1.4.26	How many other people are employed by the business, that are not usually at the shop?<br><br>NOTE FOR INTERVIEWER: If no other employees, enter "0"	Number [Allow only numbers]
1.4.27	[If 1.4.26>0] What percent of these other employees (who do not usually work at the shop) are male?	% Male
1.4.29	From how many different sources do you regularly purchase products to sell in your shop, in total? A "source" could be a supplier, a wholesaler, another market, etc. Do not include your own farm production.	Number [Allow only numbers]
1.4.29a	If 1.4.29 >0 What type of suppliers do you usually buy from?	1. Producers or farmers<br>2. Wholesalers<br>3. Middlemen<br>77. Other (specify)
1.4.29b	Approximately what proportion of key	- None (~0%)<br>- A very small/occasional part (<15%)

<table>
<tr><td></td><td>commodities is produced by you or your household?</td><td>- Some but not a lot (16-49%)
- About half (~50%)
- A large part (51-80%)
- All or almost all (81-100%)</td></tr>
<tr><td>1.4.31</td><td>How often do you get new batches of [Pipe in items at 1.4.9, response is per item] from suppliers?</td><td>[Pipe in items at 1.4.9, response is per item]
<table>
<tr><th>Item from 1.1.9</th><th>Number of times (integer)</th><th>Time unit (select one)</th></tr>
<tr><td></td><td></td><td>Week / Month / Year</td></tr>
<tr><td></td><td></td><td>Week / Month / Year</td></tr>
<tr><td></td><td></td><td>Week / Month / Year</td></tr>
<tr><td></td><td></td><td>Week / Month / Year</td></tr>
</table></td></tr>
<tr><td>1.4.32</td><td>Who primarily brings the food to your shop at [pipe-in response at 1.1.6]?</td><td>1. Self (vendor)
2. Fellow shop staff/employee
3. Supplier
77 Other (specify)
4. NA</td></tr>
</table>

MODULE 2: Vendor practices and behaviors at the market

**MODULE 2.1: Vendor choices and behaviours related to the market**		
**INTERVIEWER SAY**: I am now going to ask you more in details about what you do when working at the market, and your experiences selling food at the [pipe-in response at 1.1.6]. This second section will take approximately 10 minutes.		
	Time stamp	hr:min
**Q#**	**Question**	**Answer**
2.1.1	Why do you sell food at [Pipe-in response at 1.1.6], as opposed to selling at some other market? List up to three reasons, in order of importance.<br><br>[Scripter: ensure answers appear in the data in the same order they are selected]	1 Location<br>2 Revenues<br>3 Credit<br>4 Security<br>5 Market cleanliness<br>6 Infrastructure<br>7 Market management<br>8 Shop expenses<br>9 Product availability<br>10 Customer flow<br>77 Other (specify)<br>88 No reason
2.1.3_pre	If you were unable to sell produce at this market on a given day, for any reason, would you sell your products somewhere else?	1 Yes<br>0 No
2.1.4	Overall, how satisfied are you about your experience selling food at [Pipe-in response at 1.1.6]?	1 Not at all satisfied<br>2 Slightly dissatisfied<br>3 Neither satisfied nor dissatisfied<br>4 slightly satisfied<br>5 Completely satisfied
2.1.5	[ask if code is 1 or 2 or 3 at 2.1.4] For what reasons, if any, has your experience been less than satisfactory at [Pipe-in response at 1.1.6]? is there anything that needs improvement?	[Open-ended]

2.1.6	How personally secure do you usually feel while selling food at this market? Secure is intended here as physical or personal safety, i.e. not being worried about being harassed, or about crimes such as theft or assault.<br><br>**READOUT SCALE**	1 Very insecure<br>2 Insecure<br>3 Neither secure nor insecure<br>4 Secure<br>5 Very secure
~~2.1.6b~~	~~Is there Market management in the Market? (Market management _____)~~	~~1. Yes~~<br>~~2. No~~<br>~~If no go to Q 2.1.7~~
2.1.7	How frequently, if at all, do you talk with any officer or authority that is responsible for [Pipe-in response at 1.1.6] market?	1 Daily or almost<br>2 Weekly (at least once a week or more)<br>3 Monthly (at least once a month or more)<br>4 Every 2-3 months<br>5 Occasionally (around 2-3 times/year or less)<br>77 Other (specify)<br>6. No interaction
2.1.10	For what reasons, if any, do you talk with the any officer or authority that is responsible for ?<br>Skip if code 6 is selected on 2.1.7	1. Paying rent/taxes<br>2. Obtaining/renewing business license<br>3. Organizational meetings<br>4. Authority performing services<br>5. Checking/enforcing regulations<br>6. Requesting help<br>7. Filing complaint<br>8. Requesting information<br>9. Socializing<br>77. Other (specify)
~~2.1.11~~	~~[If No interaction in 2.1.9 What are the main reasons you do not interact with the MM?~~	[Open-ended]
~~2.1.12~~	~~What information, if any, does the MM provide you with? List all the types of information that come to mind.~~	[List]
~~2.1.13~~	~~What information or services does the Market management provide you with, if any, specifically regarding food~~	[List]

	~~quality or safety? List all the information and services that come to mind.~~	
2.1.7a	Thinking of your experience at [Pipe-in response at 1.1.6], on a scale from 1=very dirty to 5=very clean, how would you rate [pipe-in response at 1.1.6] in terms of cleanliness?<br><br>[Interviewer: if asked, explain full scale. 1= very dirty, 2=dirty, 3 = average; not too clean, but not too dirty either, 4 = clean;5 = very clean]	1 Very dirty<br>2 Dirty<br>3 Neutral<br>4 Clean<br>5 Very clean
2.1.8	[If code 1 or 2 or 3 in 2.1.7a] what makes this market dirty?	[Open-ended]
2.1.2	[If 2.1.1 NOT 5] Is the market's cleanliness ever a reason for choosing which market to sell at?	1 Yes<br>0 No

**MODULE 2.2: Vendor interactions with customers**		
**INTERVIEWER SAY**: These next questions are about your customers and how you interact with them, at [Pipe-in response at 1.1.6].		
**Q#**	**Question/Tambaya**	**Answer/Amsa**
2.2.1	When you are selling at [Pipe-in response at 1.1.6], approximately how many customers do you serve in a typical day, not the main market day?	[Number]<br>[NA/Don't Know option (exclusive)]
2.2.1b	When you are selling at [Pipe-in response at 1.1.6], approximately how many customers do you serve in a typical main market day?	[Number]<br>[NA/Don't Know option (exclusive)]
2.2.2	Of the [Pipe-in response from 2.2.1b] customers who visit your shop on a typical day, how many would you say are regular/familiar customers that often shop from you?	[Number]
2.2.3	What do you do to make it more likely that your customers purchase from you again in the future? List all the actions that come to mind.	[List]

2.2.4	Other than price, what food characteristics do customers most often seek information about? List up to three characteristics that you think are most important to customers.	[List]
2.2.5	What are the characteristics of food, if any, that customers most often complain about? List up to three in the order of importance to you.<br><br>[Scripter: re-order or rank responses based on selection]	1. Quality<br>2. Shelf Life<br>3. Food made customer or HH member sick<br>4. Taste<br>5. Price<br>6. Household satisfaction<br>7. Error in type or amount of food<br>8. None<br>77. Other (specify)
2.2.6	How often do your customers ask where you get the food from?	1 Never<br>2 Occasionally/rarely<br>3 Sometimes<br>4 Often<br>5 Every time or almost
2.2.7	What aspects of the origin or handling of the food, if any, do your customers asked about most frequently?	[Open-ended]<br>none
**INTERVIEWER SAY:** The next questions are about specific interactions with customers in the last year, while selling food at the [Pipe-in response at 1.1.6].		
2.2.8	In the last year, how frequently have you had conversations with your customers about the safety of food you sold?	1 Never<br>2 Occasionally/rarely<br>3 Sometimes<br>4 Often<br>5 Every time or almost
2.2.9	How likely are you to start (i.e. be the initiator of) a conversation with a consumer about the safety of foods?	1 Very unlikely<br>2 Unlikely<br>3 Neutral<br>4 Likely<br>5 Very likely
2.2.10	In conversations with customers about food safety, what specific foods do you most often talk about?	[Select from Key Foods listed in Table 1]<br>None
2.2.11	If 2.2.10 is none skip to 2.2.12<br>What topic(s) related to the safety of food did you most often discuss, in these conversations with consumers ?	[Open-ended]

2.2.12	Thinking specifically of whether a food is safe or unsafe to eat, how would you define "safety"?	[Open-ended]
2.2.13	From your perspective, what do you think are signs that a batch of food might not be safe? List all the signs that come to mind.	[List]

**MODULE 2.3: Vendor interactions with suppliers, and behaviours related to choosing foods from suppliers.**		
**INTERVIEWER SAY**: I am now going to ask you about suppliers you buy food from. All questions refer to the food you sell at [Pipe-in response at 1.1.6].		
**Q#**	**Question**	**Answer**
2.3.1	How frequently do you compare different suppliers before deciding where to buy?	1-Never<br>2- Occasionally/rarely<br>3-sometimes<br>4-often<br>5-Every time or almost
2.3.2	What characteristics of a supplier would make you want to purchase from them again in the future? List up to 3 most important characteristics.<br><br>**Note for interviewer: record answers in the order they are given. Do not ask the respondent to rank their answers. Allow only three answers**.<br><br>[Scripter: ensure answers appear in the data in the same order they are selected – Allow only three]	1 Prices<br>2 Food quality (other than cleanliness/ safety)<br>3 Food cleanliness/safety<br>4 Food Variety<br>5 Credit<br>6 Supplier personality/how they treat me as their customer<br>7 Comfort/Security<br>8 Trust in supplier<br>9 Personal/Family Connection<br>10 Consistent food availability<br>11 Additional services they offer (specify)<br>12 Proximity/convenience<br>77-Other (specify)
2.3.3	Considering the suppliers you purchase from, what percentage of these suppliers would you consider yourself a "regular customer" of, meaning you buy from them very often or nearly every time you purchase products to sell?	Percent

2.3.3a	[If answer to 2.3.3 is >0%] Compared to other suppliers, what do your regular suppliers do or have that makes you prefer them to others? [Select all that apply and include verbatim any answer not in pre-scripted options; as always, do not read answers aloud]	1 Prices<br>2 Food quality (other than cleanliness/ safety)<br>3 Food cleanliness/safety<br>4 Food Variety<br>5 Credit<br>6 Supplier personality/how they treat me as their customer<br>7 Comfort/Security<br>8 Trust in supplier<br>9 Personal/Family Connection<br>10 Consistent food availability<br>11 Additional services they offer (specify)<br>12.Proximity/convenience<br>77-Other (specify)
2.3.4	What were the reasons, if any, that you have stopped buying food from a specific supplier in the past? List up to three reasons.<br><br>[Scripter: ensure answers appear in the data in the same order they are selected]	1 Prices<br>2 Food quality (other than cleanliness/ safety)<br>3 Food cleanliness/safety<br>4 Food Variety<br>5 Credit<br>6 Supplier personality/how they treat me as their customer<br>7 Comfort/Security<br>8 Trust in supplier<br>9 Personal/Family Connection<br>10 Consistent food availability<br>12 Additional services they offer (specify)<br>10. Proximity/convenience<br>77-Other (specify)88 Not Applicable/Never Stopped
2.3.5	If you wanted to change suppliers, for any reason, are there other viable suppliers that you could purchase from?	1 Yes<br>0 No<br>99 Don't Know/Not Sure
2.3.6	How often do you have conversations with suppliers about the quality or safety of a food?<br>**READOUT SCALE**	1-Never<br>2- Occasionally/rarely<br>3-Often<br>4-Very often<br>5-Every time or almost

**MODULE 2.4: Vendor practices relevant to food safety**

INTERVIEWER SAY: I am now going to ask you some questions about selling food at the [Pipe-in response at 1.1.6]. They all refer to the timeframe of the last year, that is approximately from June 2021 to now (July 2022).

Q#	Question	Answer
2.4.1	What cleaning operations do you perform throughout the day while working at your shop? List all that come to mind.	[List]
2.4.3	In a typical main market day,, approximately how many times do you wash your hands while on duty?	Number [Allow only numbers]
2.4.4	What do usually you do with the food that you haven't sold that day?<br>Select all that apply	1-Store at shop for the next day<br>2-Sell outside the market the same day<br>3-Store at my home<br>4-Give it away<br>5-Discard it<br>6-Feed it to animals<br>7-Nothing<br>99-Don't know<br>77-Other (specify)
2.4.5	Throughout the day, what do you do to keep the food clean? By "clean", we mean that the food is protected from germs or other contaminants that can get into the food while at the market. List all actions that come to mind.	[List]
2.4.6	Throughout the day, what do you do to keep the food fresh? List all actions that come to mind.	[List]

INTERVIEWER SAY: These next questions are about specific actions related to selling food at the [Pipe-in response at 1.1.6].They all refer to the timeframe of the last year, that is approximately from June 2021 to now (July 2022).

2.4.7	Which changes, if any, have you made to your shop or to the way you sell food, in the past year?	- [Open-ended]<br>- No changes made
2.4.8	[If any changes mentioned in 2.4.7]<br><br>What are the reasons you made these changes?	OPEN-ENDED
2.4.9	Which tools or equipment to protect food or keep it clean or safe have you acquired in the past year, if any? Please include NEW tools/equipment and also tools or equipment that you routinely acquire and have CONTINUED to acquire in the past year. List all items that come to mind.	[List up to 6 tools or pieces of equipment or NONE]

MODULE 3: Vendor perceptions and attitudes

**MODULE 3.1: Beliefs and attitudes on vending at MARKET and MARKET MANAGEMENT**		
**INTERVIEWER SAY**: We are more than half-way through the survey. The next questions are about how you views on how [Pipe-in response at 1.1.6] is managed and operates.		
**Q#**	**Question/**	**Answer/**
**Beliefs**		
**INTERVIEWER SAY**: I'm going to read you a few statements and would like to know whether you agree or disagree with the statement, based on your own perspective, on a 1-5 scale, from 1-Strongly disagree, 2-Disagree, 3-Neither agree nor disagree, 4-Agree, 5-Strongly agree. For example, if the statement is "I like to eat fish," my personal response might be "strongly agree" if I really like eating fish, or my response might be "neither agree nor disagree" if I sometimes eat fish but don't like it a lot, and so on.		
3.1.12	"At [Pipe-in response at 1.1.6], some vendors sell food that is safer to eat than other vendors."	1 Strongly disagree<br>2 Disagree<br>3 Neither agree nor disagree<br>4 Agree<br>5 Strongly agree
3.1.13	"Some vendors at [Pipe-in response at 1.1.6] care more about food safety than others."	1 Strongly disagree<br>2 Disagree<br>3 Neither agree nor disagree<br>4 Agree<br>5 Strongly agree
3.1.14	"I trust that other vendors at [Pipe-in response at 1.1.6] will help me if I need help with doing something at the shop"	1 Strongly disagree<br>2 Disagree<br>3 Neither agree nor disagree<br>4 Agree

		5 Strongly agree
3.1.15	[If 3.1.14=1 or 2] What are the reasons you don't trust that they will help you	[Open-ended]
3.1.16	"I trust other vendors to be honest in how they conduct their business"	1 Strongly disagree<br>2 Disagree<br>3 Neither agree nor disagree<br>4 Agree<br>5 Strongly agree
3.1.17	"I don't think it is the responsibility of officer or authority that is resposible to the market to ensure the food sold at the market is safe"	1 Strongly disagree<br>2 Disagree<br>3 Neither agree nor disagree<br>4 Agree<br>5 Strongly agree
3.1.18	"The officer or authority that is responsible to the market does a good job ensuring that the market is kept clean"	1 Strongly disagree<br>2 Disagree<br>3 Neither agree nor disagree<br>4 Agree<br>5 Strongly agree
3.1.19	"The officer or authority makes sure that the food sold by vendors is safe to eat."	1 Strongly disagree<br>2 Disagree<br>3 Neither agree nor disagree<br>4 Agree<br>5 Strongly agree
3.1.20	"The officer or authority that is responsible to the market helps vendors when vendors have a problem related to their shop"	1 Strongly disagree<br>2 Disagree<br>3 Neither agree nor disagree<br>4 Agree<br>5 Strongly agree
3.1.21	"The officer or authority that is responsible to the market provides services that are useful to me"	1 Strongly disagree<br>2 Disagree<br>3 Neither agree nor disagree<br>4 Agree<br>5 Strongly agree
3.1.23	"The officer or authority that is responsible to the market helps vendors to improve or upkeep their shop structures"	1 Strongly disagree<br>2 Disagree<br>3 Neither agree nor disagree<br>4 Agree<br>5 Strongly agree
3.1.24	"The officer or authority that is responsible of the market ensures that vendors respect regulations"	1 Strongly disagree<br>2 Disagree<br>3 Neither agree nor disagree<br>4 Agree<br>5 Strongly agree

3.1.26	"For issues that the officer or authority that is responsible to the market is responsible for, I would prefer to interact with female market management representatives"	1 Strongly disagree<br>2 Disagree<br>3 Neither agree nor disagree<br>4 Agree<br>5 Strongly agree
3.1.27	"It is important to me that the government enforces acceptable standards of food quality"	1 Strongly disagree<br>2 Disagree<br>3 Neither agree nor disagree<br>4 Agree<br>5 Strongly agree
3.1.28	In general, who do you think should be primarily responsible for ensuring that the food that is sold at the market is safe? Name up to 3 in order of importance to you.<br><br>[Scripter: re-order or rank responses based on selection; four selections required]	[Open-ended]

**MODULE 3.2: Beliefs, perceptions, attitudes related to CUSTOMERS**		
**INTERVIEWER SAY**: These next questions are about how you view customers that buy food from you at [Pipe-in response at 1.1.6]		
**Q#**	**Question**	**Answer**
**Beliefs**		
3.2.1	In your opinion, what are the most important reasons that a customer might choose to come back to a vendor's shop for future purchases? List up to 3 reasons in order of importance to you.<br><br>[Scripter: ensure answers appear in the data in the same order they are selected]	[List]
I'm now going to read you a few statements related to selling food and interacting with customers at [Pipe-in response at 1.1.6] and would like to know whether you agree or disagree with the statement using the same scale as before: a 1-5 scale from 1. strongly disagree to 5. strongly agree). [Interviewer: repeat full scale if respondent asks]		
3.2.2	"My customers tell me when they are satisfied with the food they buy from me"	1 Strongly disagree<br>2 Disagree<br>3 Neither agree nor disagree<br>4 Agree<br>5 Strongly agree

3.2.3	"It is helpful/useful when a customer tells me that they are not happy with the food they purchased from me"	1 Strongly disagree<br>2 Disagree<br>3 Neither agree nor disagree<br>4 Agree<br>5 Strongly agree
3.2.4	"I get upset if a customer complains about the food they bought from me."	1 Strongly disagree<br>2 Disagree<br>3 Neither agree nor disagree<br>4 Agree<br>5 Strongly agree
3.2.5	"My customers' requests push me to improve how I operate my shop"	1 Strongly disagree<br>2 Disagree<br>3 Neither agree nor disagree<br>4 Agree<br>5 Strongly agree
3.2.6	"I take time to answer the questions that my customers have about the food I sell"	1 Strongly disagree<br>2 Disagree<br>3 Neither agree nor disagree<br>4 Agree<br>5 Strongly agree
3.2.7	"I am willing to put in extra effort in order to give my customrs exactly what they request"	1 Strongly disagree<br>2 Disagree<br>3 Neither agree nor disagree<br>4 Agree<br>5 Strongly agree
3.2.8	"I am more comfortable talking to customers of my gender than the opposite gender"<br><br>[Note: a neutral answer (3) means the respondent is equally comfortable talking to either gender]	1 Strongly disagree<br>2 Disagree<br>3 Neither agree nor disagree<br>4 Agree<br>5 Strongly agree
3.2.9	"Female customers make more requests about the food they want to purchase than male customers"<br><br>[Note: a neutral answer (3) means the respondent believes there is no difference by gender]	1 Strongly disagree<br>2 Disagree<br>3 Neither agree nor disagree<br>4 Agree<br>5 Strongly agree
**Attitudes**		
**INTERVIEWER SAY**: The next questions will ask you to rate certain characteristics based on how important they are to you. As before, we'll use a 1-5 scale, where 1= very low importance, 2= low importance, 3= moderate importance, 4= high importance, and 5= very high importance.		

3.2.10	How important is it to you that customers express questions and concerns related to the food you sell? Is it of...?	1 Very Low Importance<br>2 Low Importance<br>3 Moderate Importance<br>4 High Importance<br>5 Very High Importance
3.2.11	How important is it to you that customers can see you actively taking care of the shop? Is it of...?	1 Very Low Importance<br>2 Low Importance<br>3 Moderate Importance<br>4 High Importance<br>5 Very High Importance
3.2.12	How important is it to you that you look neat and tidy when on duty? Is it of...?	1 Very Low Importance<br>2 Low Importance<br>3 Moderate Importance<br>4 High Importance<br>5 Very High Importance
3.2.13	How important is it to you that your customers can see how food is handled in your shop? Is it of...?	1 Very Low Importance<br>2 Low Importance<br>3 Moderate Importance<br>4 High Importance<br>5 Very High Importance
3.2.14	How important is it to you that your customers be told where the food you buy comes from? Is it of...?	1 Very Low Importance<br>2 Low Importance<br>3 Moderate Importance<br>4 High Importance<br>5 Very High Importance

**MODULE 3.3: Beliefs, perceptions, attitudes related to SUPPLIERS and FOOD CHOICE**		
**INTERVIEWER SAY**: These next questions are about your perspective on choosing where you buy the food you sell at [Pipe-in response at 1.1.6]. We will ask you about "suppliers," by which we mean all the people and sources from which you get food to sell at your shop, aside from any products you produce yourself.		
**Q#**	**Question**	**Answer**
**Beliefs**		
**INTERVIEWER SAY**: I'm going to read you a few statements and would like to know whether you agree or disagree with the statement, using the same scale as before: a 1-5 scale from 1= strongly disagree to 5= strongly agree.		
3.3.3	"I am confident that I can find a supplier that sells high-quality foods." QUALITY includes safety as well as other characteristics).	1 Strongly disagree<br>2 Disagree<br>3 Neither agree nor disagree<br>4 Agree<br>5 Strongly agree
3.3.4	"When buying food from suppliers, I am confident that I know how to choose foods that are safe"	1 Strongly disagree<br>2 Disagree<br>3 Neither agree nor disagree<br>4 Agree

		5 Strongly agree
3.3.5	"I am confident that I can find a supplier that sells safe foods" Here, we are considering safety ONLY, as an aspect of quality.	1 Strongly disagree<br>2 Disagree<br>3 Neither agree nor disagree<br>4 Agree<br>5 Strongly agree
3.3.6	"When buying food, I am willing to spend a bit more time looking for the best food or shop, if I know the food I eventually get is safer."	1 Strongly disagree<br>2 Disagree<br>3 Neither agree nor disagree<br>4 Agree<br>5 Strongly agree
3.3.7	"When buying food, I am willing to spend a bit more money to get a food that I know is safe, compared to food that I'm not sure if it's safe."	1 Strongly disagree<br>2 Disagree<br>3 Neither agree nor disagree<br>4 Agree<br>5 Strongly agree
3.3.12	How satisfied are you overall about the food you buy from your suppliers?	1 Very Unsatisfied<br>2 Somewhat Unsatisfied<br>3 Neither Satisfied nor Unsatisfied<br>4 Somewhat Satisfied<br>5 Very Satisfied
3.3.13	What factors related to the food bought from your suppliers are you most satisfied with? List up to 3 in order of their importance to you.<br><br>[Scripter: ensure answers appear in the data in the same order they are selected]	1. Quality<br>2. Variety<br>3. Price<br>4. Safety<br>5. Healthiness<br>6. Cleanliness<br>7. I know where the food is from<br>8. Quantity<br>9. Household satisfaction<br>77. Other (specify)
3.3.14	What factors related to the food bought from your suppliers are you least satisfied with? List up to 3 in order of their importance to you.<br><br>[Scripter: re-order or rank responses based on selections]	1. Quality<br>2. Variety<br>3. Price<br>4. Safety<br>5. Healthiness<br>6. Cleanliness<br>7. I know where the food is from<br>8. Quantity<br>9. Household satisfaction<br>77. Other (specify)
3.3.18	If you are feeling worried that a food provided by your supplier might not be safe, what action(s) might you take to	[List]

	resolve the issue? List all actions that come to mind.	

**MODULE 3.4: Beliefs, perceptions, attitudes related to FOOD HANDLING and SHOP PRACTICES**		
**INTERVIEWER SAY**: These next questions are about your opinions and motivations for how you operate your shop at [Pipe-in response at 1.1.6].		
**Q#**	**Question**	**Answer**
**Beliefs**		
**INTERVIEWER SAY**: I'm going to read you a few statements and would like to know whether you agree or disagree with the statement (on the same scale as before, from 1-Strongly disagree to 5-Strongly agree). [Interviewer: repeat full scale if respondent asks or seems confused]		
3.4.1	"I am satisfied with how my shop looks, compared to the shops of other vendors"	1 Strongly disagree<br>2 Disagree<br>3 Neither agree nor disagree<br>4 Agree<br>5 Strongly agree
3.4.2	"I am proud of the quality of the food I sell"	1 Strongly disagree<br>2 Disagree<br>3 Neither agree nor disagree<br>4 Agree<br>5 Strongly agree
3.4.3	"I am satisfied with how my shop operates"	1 Strongly disagree<br>2 Disagree<br>3 Neither agree nor disagree<br>4 Agree<br>5 Strongly agree
3.4.6	"There are set rules/expectations in my shop for preserving the quality and safety of the food I sell."	1 Strongly disagree<br>2 Disagree<br>3 Neither agree nor disagree<br>4 Agree<br>5 Strongly agree
3.4.7	"There are set rules/expectations in my shop about how to keep the shop clean."	1 Strongly disagree<br>2 Disagree<br>3 Neither agree nor disagree<br>4 Agree<br>5 Strongly agree
3.4.8	"Sometimes it is difficult to keep the shop clean."	1 Strongly disagree<br>2 Disagree<br>3 Neither agree nor disagree<br>4 Agree<br>5 Strongly agree
3.4.9	[If 3.4.8=4 or 5] What makes it difficult to keep the shop clean?	[Open-ended]

**Attitudes: Vendor-to-Vendor Peer Interactions**		
3.4.14	How often do you talk to other vendors for help in making selling/management decisions?	1. Never<br>2. Occasionally/Rarely<br>3. Sometimes<br>4. Often<br>5. Every time or almost
**INTERVIEWER SAY**: The next questions will require you to rate certain characteristics on a scale of importance, from "Very Low Importance" to "Very High Importance"		
3.4.15	How important is it to you that you can be a good role model for other vendors? Is it of...?	1 Very Low Importance<br>2 Low Importance<br>3 Moderate Importance<br>4 High Importance<br>5 Very High Importance
3.4.16	How important is it to you that you can exchange knowledge and skills related to selling food with other vendors? Is it of...?<br>**READOUT SCALE**	1 Very Low Importance<br>2 Low Importance<br>3 Moderate Importance<br>4 High Importance<br>5 Very High Importance
3.4.17	How important is it to you that you can work together with or share responsibilities with other vendors? Is it of...?<br>**READOUT SCALE**	1 Very Low Importance<br>2 Low Importance<br>3 Moderate Importance<br>4 High Importance<br>5 Very High Importance

**MODULE 3.3: Health concerns**		
The next short series of questions, which concludes this section, is about health concerns.		
**Q#**	**Question**	**Answer**
3.5.3	Which food-borne diseases or issues, if any, are you most worried about for your household? Name up to three.	[Open-ended]
LOOP the following two questions (3.5.4 and 3.5.5) for each food-borne disease specified in 3.5.3:		
3.5.4	[For each concern selected in 3.5.3] Which food(s) are associated with this food-borne disease or issue? Name upto three.	[Select from Key Foods listed in Table 2]
3.5.5	[For each food selected in 3.5.4] Why do you think [food selected in 3.5.4] is associated with [foodborne illness selected in 3.5.3]? Select all that apply	77. Microbial contamination<br>78. Chemical contamination<br>79. Dirt/Filth<br>80. Processing methods<br>81. Preparation methods<br>82. Other (specify)

END LOOP		
3.5.6	Have you or someone in your HH experienced serious sickness related to consuming a particular food in the past year? By "seriously sick", we mean that it was difficult or impossible to work or perform daily activities.	1 Yes<br>0 No<br>99. Don't Know/Not Sure
3.5.7	Have you or someone in your HH experienced serious sickness related to drinking water in the past year?	1 Yes<br>0 No<br>99. Don't Know/Not Sure

Module 4

MODULE 4.1: Sources of information		
Module Start Time: HH:MM		
**INTERVIEWER SAY: We are three quarters of the way through the survey.** The next questions relate to what sources of information you access, and your use of media		
**Q#**	**Question**	**Answer**
4.1.1	Who do you trust to provide reliable information about health issues? (select all that apply)	1. Medical professional (doctor nurse)<br>2. Community member<br>3. Community health worker<br>4. Family member<br>5. Religious leader<br>6. Community leader<br>7. News paper<br>8. Experts on radio or TV<br>77. Other (specify)
4.1.2	Suppose you wanted to find out if the food you eat is safe. Would you consult [source] for information?<br>**Yes/No for each source, asked aloud**	1. Friends or family<br>2. Medical professionals, such as your local doctor or nurse.<br>3. Experts on Newspapers, television, or radio;<br>4. Journalists/show hosts on Newspapers, television, or radio<br>5. The internet/social media<br>6. Bureau of Trade and Industry/Health<br>7. The packaging or label on the food<br>8. A famous person you like<br>9. Local religious leaders<br>77. Other (specify)
4.1.4a	Can you name a specific issue regarding food that you wanted to know more about, or a question you had, in the last year?	1 Yes<br>2 None<br><br>4.1.4b. If yes,<br>Open-ended
4.1.5	[If 4.1.4a=1] Specifically, was there anything about food being safe or unsafe, that you wanted to know more about?	1 Yes<br>0 No<br>4.1.5b. If yes, what was the topic you were interested in?<br>Open-ended

4.1.6	[If 4.1.5=1] If you looked for that information, which resources or people did you lseek out?<br>(Select all that apply)	1. Friends or family<br>2. Medical professionals, such as your local doctor or nurse.<br>3. Experts on Newspapers, television, or radio; Journalists/show hosts on Newspapers, television, or radio the internet/social media<br>4. Bureau of Trade and Industry/Health<br>5. The packaging or label on the food<br>6. A famous person you like<br>7. Local religious leaders<br>77. Other (specify)<br>88. Did not look for information

MODULE 4.2: Media usage		
**Q#**	**Question**	**Answer**
Module Start Time: HH:MM		
4.2.1	How often do you use the internet, including social media, per week (for any use)?	1 Never → **GO TO 4.2.6**<br>2 a few times per week<br>3 most days but not every day<br>4 every day
4.2.2	On what device(s) do you usually access Internet? List all applicable devices.	1 Smartphone<br>2 Mobile Tablet<br>3 Laptop Computer<br>4 Desktop Computer77Other (specify)
4.2.2_post	[For each selection in 4.2.2]<br><br>Do you personally own one or more of this type of device?	1 Yes<br>0 No
4.2.3	Where do you usually access internet? List all applicable places.	1 Home<br>2 On my phone, wherever I am<br>3 Friend's, Relative's, or Neighbor's Home<br>4 Internet Café<br>5 Public Library<br>77 Other (specify)
4.2.4	What social media platforms do you regularly use, if any?	1 Facebook<br>2 Twitter<br>3 YouTube<br>4 WhatsApp<br>5 Instagram<br>6 Telegram<br>7 Tik Tok<br>8 None<br>77 Other (specify)
4.2.6	Which media channel do you use to get entertainment? By entertainment we mean that it brings amusement, enjoyment, or relaxation (not to obtain information).	1 Normal TV<br>2 Satellite TV<br>3 Radio<br>4 Movie theatre/cinema<br>5 Internet<br>6 Social media<br>7 Messaging apps<br>77. Other (specify)

4.2.7a	For each of the channels selected, how often do you use them?<br>[Pipe-in selection from 4.2.6 and apply response as drop-down per selection]	1-daily<br>2-2 or 3 times per week<br>3-once a week<br>4-once every two weeks<br>5-once per month<br>6. less than once per month
4.2.8	When you are looking for entertainment, what content do you most often look for?<br><br>(select all that apply)	1. TV shows, films/series/soap operas,<br>2. Radio serials,<br>3. Online videos,<br>4. Social media,<br>5. In-person word of mouth<br>77. Others specify
Module End Time: HH:MM		

[Survey Closing]: We are at the end for the survey. Thank you very much for sharing your valuable opinion. The information received from you and from other participants will be very useful in making food safer at the markets. To express our appreciation, we would like to give you a thank you token of ________birr for your time you spend with us. i
This study will conduct other surveys in the next two years. Could we contact you again at that time?
1. Yes
2. No

1.1.5b	Interview end time	hr:min (in 24 hr notation)
