# Supplementary material for: Food Safety Practices and Behavior Drivers in Traditional Food Markets in Ethiopia: Assessing the Potential for Consumer-Driven Interventions
Source: Int J Environ Res Public Health. 2025 Oct 29;22(11):1645. doi: 10.3390/ijerph22111645 (PMC12652902; doi:10.3390/ijerph22111645)
Supplement: Supplementary file 1 [file ijerph-22-01645-s001.zip › S3_PPI scoring.pdf]

**Food Safety Practices and Behavior Drivers in Traditional Food Markets in Ethiopia: assessing the potential for consumer-driven interventions**

*Ariel V. Garsow, Smret Hagos, Eric Djimeu, Carrel Fokou, Haley Swartz, Genet Gebremedhin, Bisaku Chacha, and Elisabetta Lambertini*

**5.3 Supplemental Information 3:** Ethiopia-specific PPI indicators and corresponding scores.

**Supplemental Table 1.** *Ethiopia-specific PPI indicators and corresponding scores.*

| Indicators | Responses | Points | |
|---|---|---|---|
| | | **Ethiopian NPL (7,184 ETB/day)** | **International PL ($3.20/day)** |
| In which region does the household live? | A. Amhara | 0 | 0 |
| | B. Oromiya | 9 | 9 |
| | C. SNNP | 0 | 0 |
| | D. Tigray | 5 | 3 |
| | E. Other regions | 1 | 0 |
| How many members are there in the household? | A. 1 to 4 | 22 | 20 |
| | B. 5 to 7 | 11 | 9 |
| | C. 8 or more | 0 | 0 |
| What is the highest grade that the household head completed? | A. Kindergarten | 0 | 0 |
| | B. Nursery | 0 | 0 |
| | C. 0 grade | 0 | 0 |
| | D. From 1st to 4th grade | 0 | 0 |
| | E. Fifth grade or above | 10 | 10 |
| | F. Informal education (literate, but has never been in regular school) | 10 | 10 |
| | G. Adult literacy program | 10 | 10 |
| | H. Satellite | 10 | 10 |
| | I. Non-regular (literate, but never attended regular school; attended religious institutions like Kes or Kuran) | 10 | 10 |
| | J. Illiterate (not educated) | 0 | 0 |
| | K. Never attended school | 0 | 0 |
| Over the past 7 days, did you or others in your household consume any beef? | A. Yes | 17 | 19 |
| | B. No | 0 | 0 |

| Indicators | Responses | Points | |
|---|---|---|---|
| | | **Ethiopian NPL (7,184 ETB/day)** | **International PL ($3.20/day)** |
| Over the past 7 days, did you or others in your household consume any horse beans? | A. Yes | 8 | 7 |
| | B. No | 0 | 0 |
| The roof of the main dwelling is predominantly made of what material? | A. Thatch | 0 | 0 |
| | B. Mud and Wood | 0 | 0 |
| | C. Bamboo/Reed | 0 | 0 |
| | D. Plastic Canvas | 0 | 0 |
| | E. Corrugated Iron Sheets | 3 | 3 |
| | F. Concrete/Cement | 3 | 3 |
| | G. Asbestos | 3 | 3 |
| | H. Bricks | 3 | 3 |
| | I. Other | 0 | 0 |
| What type of toilet facility does the household use? | A. PIT Latrine without slab | 0 | 0 |
| | B. Composting toilet | 0 | 0 |
| | C. Field/Forest | 0 | 0 |
| | D. Flush toilet | 4 | 3 |
| | E. PIT Latrine (ventilated pit) | 4 | 3 |
| | F. PIT Latrine with slab | 4 | 3 |
| | G. Bucket | 4 | 3 |
| | H. Other | 0 | 0 |
| What is the main source of light for the household? | A. Bio gas | 0 | 0 |
| | B. Electrical battery | 0 | 0 |
| | C. Light from dry cell with switch | 0 | 0 |
| | D. Kerosene light lamp (imported) | 0 | 0 |
| | E. Local kerosene lamp (Kuraz) | 0 | 0 |
| | F. Candle/Wax | 0 | 0 |
| | G. Firewood | 0 | 0 |
| | H. Electricity meter-private | 8 | 8 |
| | I. Electricity meter-shared | 8 | 8 |

| Indicators | Responses | Points: Ethiopian NPL (7,184 ETB/day) | Points: International PL ($3.20/day) |
|---|---|---|---|
| What is the main source of light for the household? | J. Electricity from generator | 8 | 8 |
| | K. Solar energy | 8 | 8 |
| | L. Lantern | 8 | 8 |
| | M. Other | 8 | 8 |
| What is the main source of cooking fuel? | A. Collecting firewood | 0 | 0 |
| | B. Crop residue/leaves | 0 | 0 |
| | C. Dung/Manure | 0 | 0 |
| | D. Saw dust | 0 | 0 |
| | E. Solar energy | 0 | 0 |
| | F. Biogas | 0 | 0 |
| | G. Purchased firewood | 9 | 11 |
| | H. Charcoal | 9 | 11 |
| | I. Kerosene | 9 | 11 |
| | J. Butane-Gas | 9 | 11 |
| | K. Electricity | 9 | 11 |
| | L. Solar energy | 9 | 11 |
| | M. None | 0 | 0 |
| | N. Other | 0 | 0 |
| How many mattresses does your household own? | A. Zero | 0 | 0 |
| | B. One | 6 | 7 |
| | C. Two or more | 10 | 10 |
